# Patient-specific coronary blood supply territories for quantitative perfusion analysis

**DOI:** 10.1080/21681163.2016.1192003

**Published:** 2016-07-13

**Authors:** Constantine Zakkaroff, John D. Biglands, John P. Greenwood, Sven Plein, Roger D. Boyle, Aleksandra Radjenovic, Derek R. Magee

**Affiliations:** ^a^ School of Computing, The University of Leeds, Leeds, UK; ^b^ Division of Medical Physics and Leeds Institute of Cardiovascular and Metabolic Medicine, University of Leeds, Leeds, UK; ^c^ Multidisciplinary Cardiovascular Research Centre and Leeds Institute of Cardiovascular and Metabolic Medicine, University of Leeds, Leeds, UK; ^d^ Institute of Biological, Environmental and Rural Sciences, University of Aberystwyth, Aberystwyth, UK; ^e^ Institute of Cardiovascular and Medical Sciences, British Heart Foundation Glasgow Cardiovascular Centre, University of Glasgow, Glasgow, UK

**Keywords:** Quantitative myocardial perfusion analysis, patient-specific coronary supply territories, image registration

## Abstract

Myocardial perfusion imaging, coupled with quantitative perfusion analysis, provides an important diagnostic tool for the identification of ischaemic heart disease caused by coronary stenoses. The accurate mapping between coronary anatomy and under-perfused areas of the myocardium is important for diagnosis and treatment. However, in the absence of the actual coronary anatomy during the reporting of perfusion images, areas of ischaemia are allocated to a coronary territory based on a population-derived 17-segment (American Heart Association) AHA model of coronary blood supply. This work presents a solution for the fusion of 2D Magnetic Resonance (MR) myocardial perfusion images and 3D MR angiography data with the aim to improve the detection of ischaemic heart disease. The key contribution of this work is a novel method for the mediated spatiotemporal registration of perfusion and angiography data and a novel method for the calculation of patient-specific coronary supply territories. The registration method uses 4D cardiac MR cine series spanning the complete cardiac cycle in order to overcome the under-constrained nature of non-rigid slice-to-volume perfusion-to-angiography registration. This is achieved by separating out the deformable registration problem and solving it through phase-to-phase registration of the cine series. The use of patient-specific blood supply territories in quantitative perfusion analysis (instead of the population-based model of coronary blood supply) has the potential of increasing the accuracy of perfusion analysis. Quantitative perfusion analysis diagnostic accuracy evaluation with patient-specific territories against the AHA model demonstrates the value of the mediated spatiotemporal registration in the context of ischaemic heart disease diagnosis.

## 1. Introduction

In the last decade, dynamic contrast-enhanced magnetic resonance (DCE-MR) myocardial perfusion imaging has become an important diagnostic tool for the identification of ischaemic heart disease. Ischaemia can be diagnosed through visual detection of myocardial regions with reduced blood supply in perfusion series. Accurate mapping between coronary arteries and perfusion information helps to identify culprit coronary arteries before the patient undergoes further, often invasive, testing.

**Figure 1.**
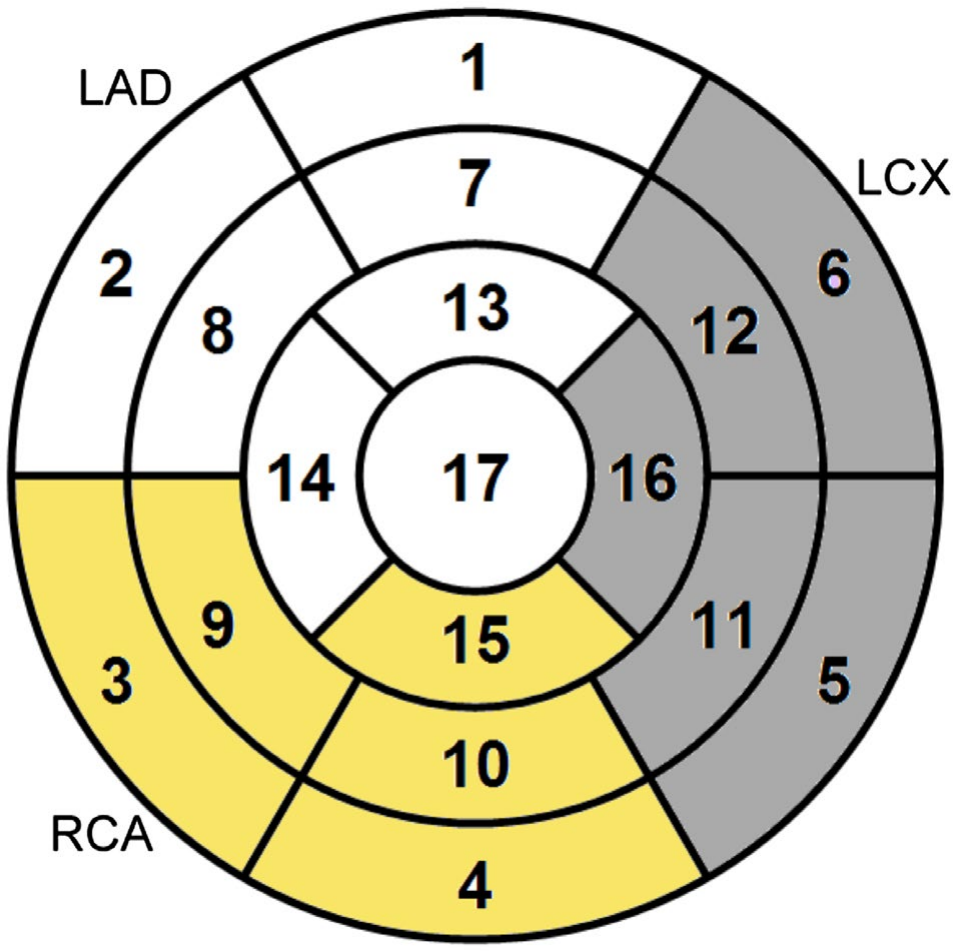
The three coronary supply territories are defined by the proximity of the segments to the main coronary arteries: LAD, RCA and LCX. For example, the LAD-supplied territory consists of segments 1, 2, 7, 8, 13, 14 and 17.

In theory, perfusion series could be used to deduce the location of the coronary stenosis according to the population-based American Heart Association (AHA) 17-segment model of coronary blood supply (Cerqueira et al. 2002): the mapping of each of the segments in the AHA model to one of the three coronary arteries is intended to establish a separate diagnosis for each coronary artery as demonstrated in Figure 1. In practice, coronary anatomy varies from patient to patient; this is acknowledged as the main limitation of the AHA model. Due to the variations in the coronary anatomy, the AHA model segmentation often results in patient-specific assignment of the myocardial segments. This can be resolved through visual analysis of X-ray angiography (Ortiz-Pérez et al. 2008; Javadi et al. 2010).

Instead of visual analysis, the correspondence between myocardial anatomy and coronary anatomy can be obtained through registration of cardiac perfusion series (and other cardiac imaging modalities) to high-resolution magnetic resonance angiography (MRA) or computed tomography angiography (CTA). The correspondence between coronary and myocardial anatomy can be used for the calculation of patient-specific coronary supply territories. While the concept of patient-specific territories is not new, to the authors’ knowledge the use of patient-specific territories has been limited to informative visualisation. The work reported in this article provides two novel contributions concerned with the fusion of cardiac image data:

• First, this article introduces a novel solution for the registration/fusion of 2D perfusion series and 3D angiography data-sets: the mediated spatiotemporal registration.
• Second, the article provides a novel method for the calculation of patient-specific mappings of coronary supply territories. Those can be used directly in quantitative perfusion image analysis.

**Figure 2.**
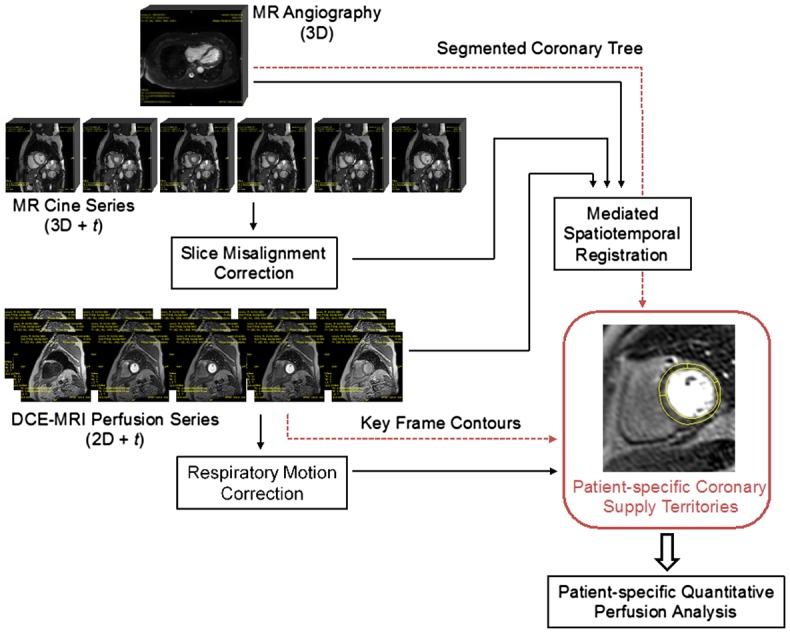
Overview of the process for the calculation of patient-specific coronary supply territories: the mediated spatiotemporal registration provides a geometric transform required for compensating for the cardiac phase difference; when applied to the coronary tree this transform places the segments of the coronary tree into the phase-specific coordinate frame corresponding to perfusion images; the warped coronary tree and myocardial contours for the maximum contrast frame are used for calculating the coronary anatomy-based perfusion territories; the transforms obtained from respiratory motion correction stage are used to propagate the perfusion territories to the rest of perfusion series.

What follows is an overview of the remainder of this article. Section 2 reviews the approaches to the registration of myocardial perfusion series with other types of cardiac images. Section 2 reviews the approaches to calculating patient-specific coronary supply territories. Figure 2 provides a schematic overview of the steps in the pipeline for the calculation of the patient-specific coronary supply territories and the types of MR data required. Section 3 provides the details of cardiac MR data used in the experiments reported in this study. Section 4 describes the central topic of this article: the mediated spatiotemporal registration. A necessary cine series pre-processing step is slice misalignment correction described in Section 4.1, followed by the component steps of the mediated spatiotemporal registration, such as perfusion-to-cine registration with cardiac phase selection (Section 4.2), angiography-to cine registration with phase selection (Section 4.3), cine phase-to-phase registration (Section 4.4) and the combination of the transforms to obtain the composite spatiotemporal transform (Section 4.5). Section 5 presents a simplified novel approach to motion correction in myocardial perfusion series. The method for the calculation of patient-specific coronary supply territories is provided in Section 6, followed by evaluation methods in Section 7, results in Section 8 and conclusions in Section 9.

## 2. Background

An extensive project relating to the combined analysis of DCE-MRI, late gadolinium enhancement (LGE) magnetic resonance images and MRA images was reported by Hennemuth et al. (2008) where they propose a solution for fusion of DCE-MRI and LGE through the use of the high-resolution MRA reference volume. The authors use slice-to-volume registration for the alignment of maximal contrast frames from perfusion series to the MRA reference volume. The authors propose the use of the affine transform as the means of dealing with cardiac phase differences; however, the authors acknowledge that slice-to-volume registration with an affine transformation is not suitable for compensation of large cardiac phase differences. In fact, it is acknowledged in numerous publications (some of which are reviewed further in this section) that cardiac phase difference is more accurately modelled with deformable transforms.

One of the first examples of multiple cardiac MR image fusion through temporal series registration is presented in the work of Gao et al. (2004). In this work, the consecutive cine frame-to-frame registration produces vector deformation fields (VDFs) which are used to transform LGE images into a synthetic cine series. This solution is based on a non-rigid registration algorithm known as multiple scale signal matching (MSSM) (Siebert & Marshall 2000) and the Cross-Correlation image similarity metric. Consecutive frame-to-frame registration constitutes the basis of the cardiac phase difference compensation in the mediated spatiotemporal registration proposed in this work. In general, consecutive frame-to-frame registration features in multiple registration instances listed in this section.

Another example of consecutive frame-to-frame registration is provided in the work Peyrat et al. (2010). The authors propose the use of the physically constrained diffeomorphic Demons algorithm for calculation of a phase-to-phase VDFs which are used for the estimation of myocardium strain. The key relevance of this work in the context of the registration method proposed in this article is the use of a frame-by-frame registration strategy with the view of reducing registration errors arising from the changing appearance of trabeculae, papillary muscles and other objects in the images.

The work of Perperidis et al. (2005) presents a well-structured framework for the construction of a probabilistic cardiac atlas through the spatiotemporal alignment of cardiac MR cine series. In this framework, the spatiotemporal registration is modelled as a 4D deformable transformation separated into the global spatial, global temporal, local spatial and local temporal components. The global transformation components correspond to the variations in the population captured in the data-sets, while the local transformation components correspond to the variation in the shape of the registered object within one particular cine series. The mediated spatiotemporal registration solution which is the subject of this article does not require the global component of the transformation model. However, the decomposition of the local shape variations into spatial and temporal components of the transformation model and their separate optimisation are taken as the underlying idea behind the mediated spatiotemporal registration. The work of Perperidis et al. (2005) proposes the idea of registration-based phase selection where the cardiac phase match is determined by the best value of the image similarity metric calculated during the consecutive registration of the angiography volume to the dynamic frames of the cine series. Registration with a rigid transform in this case allows determination of the best phase match based on the comparison of the image similarity metric values.

A series of similar solutions for producing AHA-like bull’s eye plots (BEPs) of patient-specific blood supply territories have been reported by Beliveau et al. (2007), Termeer et al. (2010), Kirişli et al. (2012). In these solutions a coronary tree (either manually or automatically segmented in coronary tree angiography) is first projected on the epicardial surface of the left ventricle (LV) (also either manually or automatically segmented in CTA). In the next step, patient-specific territories are computed on the epicardial surface by associating each point of the surface mesh with the nearest coronary artery through the shortest geodesic distance. The final 2D polar plots with patient-specific territories are produced by ‘unfolding’ the endocardial surface of the LV (which is approximated by an ellipsoid) onto a plane. Kirişli et al. (2012) go as far as the computation of perfusion-specific parameters and colour-coded visualisations relating perfusion defects and coronary artery stenosis. The authors present an elaborate solution for fusing CTA and MR perfusion imaging. They do not, however, describe how cardiac phase difference is bridged when the patient-specific territories are mapped onto perfusion images which show myocardium slices in cardiac phases other than the cardiac phase captured in the CTA.

The method for computing patient-specific territories presented in this article accounts for phase difference between MR perfusion cardiac phase (for a given slice) and the MR angiography cardiac phase through the use of a novel form of mediated spatiotemporal registration. The need for compensating for the deformation of the coronary tree between cardiac phases is substantiated by the evidence of 3D displacement of points in the coronary tree of up to 7.4 (±4.3) mm between end-systolic and end-diastolic phases as reported by Shechter et al. (2003). Contractile movement of the myocardium includes rotation around the long axis of the LV. This needs to be accounted for when patient-specific territories are generated for perfusion slices with a cardiac phase different from the phase of the angiography image.

## 3. Image data

The MR images used in this study were acquired during a trial for clinical evaluation of MRI in coronary heart disease (CHD) diagnosis, the CE-MARC trial (Greenwood et al. 2012). The CE-MARC trial was conducted in order to establish the diagnostic accuracy of a multi-parametric MR protocol for CHD detection and to compare this against myocardial perfusion scintigraphy (by SPECT) using X-ray coronary angiography as the gold-standard. Uniquely, X-ray angiography, SPECT and MR were performed on all patients, irrespective of clinical need, minimising referral bias. The severity of coronary stenoses were calculated by quantitative coronary angiography (QCA); subsequently, the sites of stenoses in the arteries were mapped to myocardial anatomy using the 17-segment AHA model of myocardial blood supply. In total 752 patients were randomised into the CE-MARC trial. The 50 data-sets used in this study were selected by the Clinical Trials Unit at the University of Leeds to be representative of the CE-MARC trial population in terms of risk factors and disease severity. In order to obtain an unambiguous data-set, patients with borderline disease severity (X-ray angiography stenosis severity between 50 and 70% or with discordance between SPECT perfusion and X-ray angiography results) were excluded.

The MR images in the CE-MARC trial were acquired on a dedicated cardiac MR scanner (1.5 Tesla Intera CV, Philips, Best, The Netherlands). The experiments reported in this study involved three types of cardiac data from the CE-MARC trial:

• *Whole-heart coronary angiography*: Three dimensional, whole-heart coronary MRA was acquired with a respiratory navigator to compensate for respiratory motion during free breathing. Timing of the diastolic rest period was estimated from a separate four-chamber free breathing cine scan. The angiography data-sets in this study were acquired in axial orientation. Pulse sequence parameters: balanced SSFP, TE 2.3 ms, TR 4.6 ms, flip angle 100°, T2 and fat saturation pre-pulses, SENSE factor 1.7, duration of acquisition up to 120 ms per R–R interval (determined by the length of diastolic rest period), matrix 304 × 304, field of view 320–460 mm, slice thickness 0.9 mm, 100–120 slices as required.
• *Resting wall-motion*: A contiguous cine stack encompassing the entire LV in 10–12 slices (depending on left ventricular long axis length) was acquired during multiple breath-holds. Pulse sequence parameters: balanced SSFP, TE 1.7 ms, TR 3.5 ms, flip angle 60°, SENSE factor 2, matrix 192 × 192, field of view 320–460 mm, slice thickness 10 mm, with at least 20 phases per cardiac cycle and 1–2 slices acquired per breath-hold.
• *Stress and rest perfusion*: Three short axis (SA) slices (basal, medial and apical) acquired during the first pass of the contrast agent through the myocardium. The correspondence of cardiac phase within each spatial location is achieved through ECG gating. A T1-weighted saturation-recovery single-shot k-space gradient echo pulse sequence combined with SENSE. Breath-hold time was chosen to coincide with the time of arrival of the contrast agent in the LV. Pulse sequence parameters: TE 1.0 ms, TR 2.7 ms, flip angle 15°, single saturation pre-pulse per R–R interval shared over three slices, SENSE factor 2, matrix 144 × 144, field of view 320–460 mm, slice thickness 10 mm.

Although initially it was planned to include all 50 data-sets in all stages of evaluation, the creation of the ground-truth data-sets was complicated due to insufficient quality of some of the data-sets. For example, for evaluation steps based on the contour-overlap metrics, in some instances the absence of a clear blood-to-tissue interface in the left ventricular cavity prohibited the use of such data-sets. Similarly, for the final evaluation step of the diagnostic performance of the patient-specific coronary supply territories. The quality of MRA data for manual segmentation of the coronary tree was sufficient only in 18 data-sets. The criterion for MRA data-set exclusion is described in detail in Section 6.

## 4. Mediated spatiotemporal registration

The main hurdle in the registration of DCE-MRI perfusion and coronary MRA arises from the requirements of DCE-MRI acquisition which dictate that all three spatial slices are obtained during a single heartbeat. This results in a spread of cardiac phases within each dynamic perfusion frame. In the proposed registration method, the 4D resting wall-motion series (hereafter referred to as cine series) provides the means of compensating for the cardiac phase difference between the perfusion series and angiography data.

**Figure 3.**
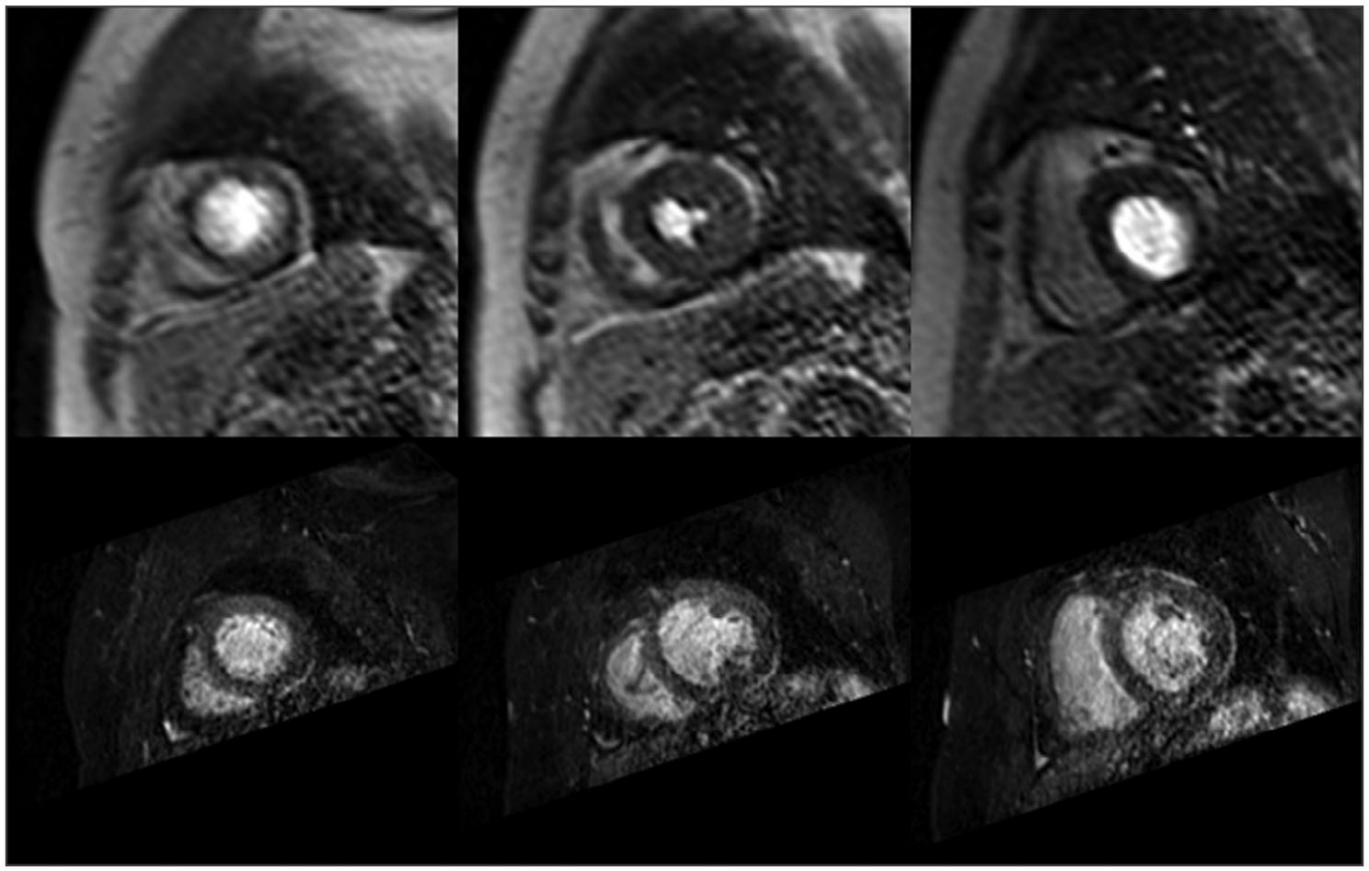
Illustration of cardiac phase differences between stress perfusion slices (top) and spatially corresponding reformatted angiography slices (bottom); all angiography slices show the myocardium in late-diastolic phase, while in perfusion slices cardiac phases are spread throughout the cardiac cycle due to the requirements of DCE-MRI protocol.

The cardiac cycle phase differences between angiography and perfusion data-sets are observed as variations of the myocaridal shape between the data-sets. The cardiac phase mismatch renders the results of slice-to-volume registration unreliable. The images in Figure 3 visualise the differences in the shape of the myocardium between angiography volumes and perfusion series.

The mediated spatiotemporal registration solution described here is designed to provide a geometric transform which would allow the mapping of 2D SA perfusion images of the myocardium acquired at either systolic or diastolic phases of the cardiac cycle to 3D angiography volumes acquired during maximum myocardial relaxation.

**Figure 4.**
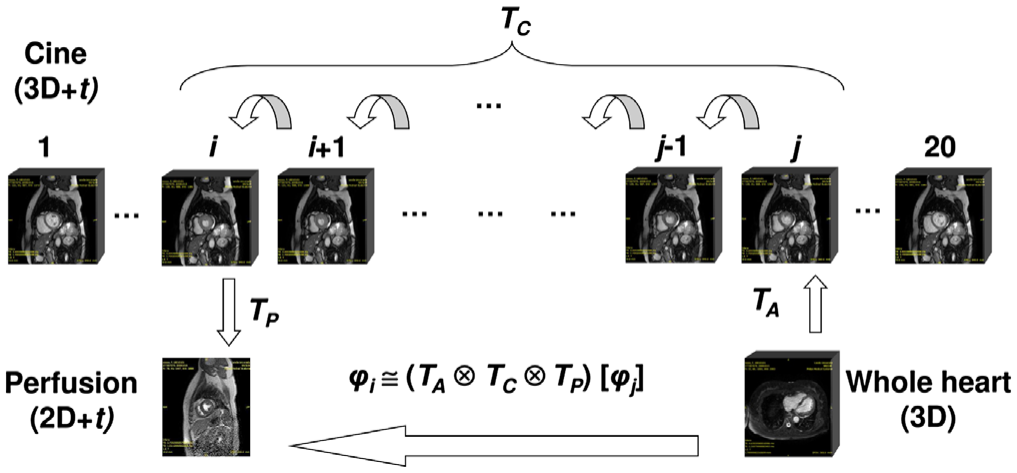
Overview of the mediated spatiotemporal registration: first, angiography-to-cine registration is carried out to obtain transform TA and phase number *p*_*j*_; next, perfusion-to-cine registration is carried out to obtain transform TP and the corresponding phase number pi; finally, cine phase-to-phase registration between phases *p*_*i*_ and *p*_*j*_ is carried out to obtain transform TC; the composition of the obtained transforms results in the whole-heart 3D angiography being registered to the maximal contrast temporal frame of the 2D perfusion series with the spatiotemporal transform which spans the cardiac phase difference between angiography and perfusion.

A schematic representation of the mediated spatiotemporal registration is provided in Figure 4. The key idea behind the mediated spatiotemporal registration is the computation of a composite transform which establishes the common spatiotemporal frame of reference for two arbitrary cardiac phases *p*_*i*_ and *p*_*j*_. These cardiac phases are implicitly defined by the perfusion and angiography images. The explicit phase correspondence is calculated in the process of registration where two cine frames *i* and *j* are selected as the cardiac cycle points matching the perfusion and angiography phases, respectively. The cine series are used in all three stages of the mediated spatiotemporal registration: perfusion-to-cine registration with phase selection, angiography-to-cine registration with phase selection and phase-to-phase transform computation which will be described in the following sections. These three stages of the mediated spatiotemporal registration produce the three transforms *T*_P_, *T*_A_ and *T*_C,_ respectively, which are combined into a final composite transform *T*_M_ for warping angiography data into a perfusion-dependent cardiac phase/coordinate space. The *T*_M_ transform is defined as follows:

(1) $$T_{\text{M}}=T_{\text{A}}⊗T_{\text{C}}⊗T_{\text{P}}.$$

The independent optimisation of the *T*_P_, *T*_A_ and *T*_C_ transforms resolves the under-constrained nature of deformable registration of 2D perfusion slices to a 3D whole-heart volume. It must be noted that although perfusion, angiography and cine images all belong to the same modality, MRI perfusion-to-angiography registration is nonetheless treated as an intermodality registration problem. This is because of the non-linear tissue/intensity mapping between perfusion, angiography and cine data. Prior to the mediated spatiotemporal registration, a pre-processing step for the correction of slice misalignment in the cine series is usually required. The following section provides a brief description of slice misalignment correction.

### 4.1. Slice misalignment correction in cine series

Slice misalignment in cine series can be observed as the displacement of individual slices in image stacks relative to each other. During cine series acquisition, breath-holding is used to exclude respiratory motion; breath-holds are applied multiple times while one to three slices over all cardiac phases are acquired during each breath-hold. Slice misalignment in cine series is caused by inconsistencies in breath-hold positions between slice acquisitions and depends on the ability of the patient to follow the instructions of the attending technician during the scan. The magnitude of individual slice displacement can vary significantly from patient to patient. In this work, slice misalignment correction in cine series was achieved with a registration method based on the simultaneous transform parameter optimisation for all cine slices in a given cine frame; this solution is based on the stack alignment transform which is a custom geometric transform designed to circumvent the issues associated with unconstrained slice-to-volume registration and preserve cine stack integrity to avoid overlaps and gaps in the corrected cine series. The details of this method for stack misalignment correction can be found in the work of Zakkaroff et al. (2012).

### 4.2. Perfusion-to-cine registration

Perfusion-to-cine registration is a problem where a 2D slice from the perfusion series is aligned to its correct spatiotemporal position within a 4D (3D + *t*) series. The optimal solution for this problem is provided with a custom spatial transform. ECG-gating is typically used for the synchronisation of image acquisition to cardiac phases, where the patient’s ECG signal is measured in order to trigger scans at the particular phase of the cardiac cycle. The ECG trigger delay is measured in milliseconds and recorded as a temporal offset after the start of the R–R interval. The absolute value of the ECG trigger delay can be used to calculate a normalised value of the cardiac phase, which depends on the patient’s heart rate during acquisition. While the ECG trigger delay normalisation can provide an approximate value for the position of the slice in the temporal dimension Φ, in many cases this value may not be optimal. In the case of the stress perfusion series, the ECG trigger delay normalisation becomes even less reliable because the variations in the patient’s heart rate cause the relative shortening and lengthening of the systolic and diastolic phases of the cardiac cycle (Umetani et al. 1999). However, ECG trigger delay normalisation is suitable for the transform initialisation prior to registration as described further in this section.

The spatiotemporal transform for perfusion-to-cine registration encapsulates a rigid centred 2D transform and augments it with displacement parameters for the third and fourth dimensions, *Z* and Φ, respectively. The out-of-plane rotations during the perfusion-to-cine registration with the spatiotemporal transform are avoided to limit the rotational errors to in-plane rotation errors. The centred rigid 2D transform includes in-plane rotation measured in radians around a centre of rotation and translation in physical space coordinates along the *X* and *Y* dimensions; the displacement parameters along the *Z* and Φ dimensions represent translations in physical and temporal coordinates. Thus the parameters to represent the spatiotemporal transform can be listed as *T* = {*θ*, *C*_*x*_, *C*_*y*_, *T*_*x*_, *T*_*y*_, *T*_*z*_, *T*_*φ*_}*.* The spatiotemporal transform does not require fixed parameters. Given a point *P* = [*x*, *y*, *z*, *φ*] in 4D space, the transformed point *P′* = [*x′*, *y′*, *z′*, *φ′*] is calculated as follows:

$$\left[\begin{array}{c} x^{\prime }\\ y^{\prime } \end{array}\right]=\left[\begin{array}{cc} \text{cos}\;\theta & -\text{sin}\;\theta \\ \text{sin}\;\theta & \text{cos}\;\theta \end{array}\right]\cdot \left[\begin{array}{c} x-C_{x}\\ y-C_{y} \end{array}\right]+\left[\begin{array}{c} T_{x}+C_{x}\\ T_{y}+C_{y} \end{array}\right]$$

$$z^{\prime }=z+T_{z}$$

(2) $$\varphi^{\prime }=\varphi +T_{\varphi }.$$

The centre of rotation parameters *C*_*x*_ and *C*_*y*_ in each case were initialised to the centre of the ROI for a given perfusion slice. The ROIs were defined manually within the basal, medial and apical slices of the key frame in rest and stress perfusion series.

The optimisation of a transform for a given 2D perfusion slice within the 4D cine series presents a challenge, because of the additional degree of freedom in the temporal dimension Φ. Furthermore, in many instances a 2D perfusion slice may match up to two likely spatiotemporal positions within the 4D cine series due to the cyclic nature of the cardiac cycle. Perfusion slices which fall before myocardial contraction at the start of the cine series may also appear to fit the end-diastolic phases of the series; and so, there might be up to two equally plausible minima for one set of transform parameters. Thus, an accurate initialisation of the spatiotemporal transform plays an important role for reliable registration. While the shape of the myocardium changes considerably throughout the cardiac cycle, the phase match between the basal perfusion slice and the corresponding cine frame is the easiest one to establish. This is because the slices of the basal locations carry the largest amount of information density compared to the medial and apical locations. Due to the cyclic nature of the cardiac motion pattern, the shape of the myocardium in a given perfusion slice can match to up to two temporal positions in the corresponding cine series, if the temporal perfusion phase is different from the maximal systolic or maximal diastolic phase. Through initial experimentation it was verified that the correct temporal location for basal slices typically lies within the window of ±3 phases centred around the phase value determined by ECG trigger delay normalisation. The use of trigger delay normalisation value for transform initialisation allows to avoid the confusion between multiple matching points in the cardiac cycle.

The importance of temporal position initialisation increases progressively for the slices closer to the apex. This is because they carry progressively smaller amounts of visual features and the optimal spatiotemporal position frequently lies further away from the phase value determined by ECG trigger delay normalisation. The optimisation of the transform parameters was achieved with a regular-step gradient descent optimizer (Ibáñez et al. 2005) and Mattes’ implementation of the mutual information (MI) (Mattes et al. 2001) image similarity metric. Image contrast changes as the contrast agent bolus passes through the myocardium. For each patient, the maximal contrast frame was identified visually as the frame where the myocardium could best be discerned from the surrounding tissues. During registration, the maximal contrast perfusion slice was used as the fixed image, while the cine series were used as the moving image during registration. In all instances the manually defined rectangular regions of interest including both ventricles were used to calculate elliptic masks to exclude irrelevant image features.

### 4.3. Angiography-to-cine registration

Similar to perfusion-to-cine registration, this registration step uses registration-based phase determined by the best MI value computed during registration for all of cine phases and angiography volume. Registration of the angiography volumes to the cine series in this case was done on a frame-by-frame basis with translation transform, regular-step gradient descent optimizer and Mattes’ implementation of the MI image similarity metric. In each registration instance, a given cine frame was used as the fixed image, while the whole-heart volume was used as the moving image in all cases. Again, manually defined ROIs for the cine series were used to derive extruded elliptical image masks to exclude irrelevant anatomical features. The best MI metric value for a given phase selected among all MI metric values obtained during registration indicated the best-matching cine/cardiac phase.

It must be noted that theoretically, if the whole-heart volume and cine series had equivalent voxel spacing, it would be possible to run registration only once (similar to the stage of perfusion-to-cine registration), with the whole-heart volume and the cine series used as the fixed and moving images, respectively. However, the voxel spacing for whole-heart volumes is much denser than in the cine series; this would result in a large number of redundant samples collected by the image similarity metric because the metric sampling is done on the image grid of the fixed image.

### 4.4. Cine phase-to-phase registration

Given the cardiac phases (corresponding to the cine frame numbers) calculated during perfusion-to-cine registration (for either of the basal, medial and apical locations), *φ*_P_, and the angiography-to-cine registration, *φ*_A_, the non-rigid 3D transform *T*_*C*_ between the *φ*_P_ and *φ*_A_ frames of the cine series is obtained through consecutive frame-to-frame 3D deformable registration of all phases from *φ*_A_ to *φ*_P_.

The implementation of the method for obtaining the phase-to-phase transform *T*_C_ presents a combination between the Lagrangian and Eulerian views of registration (Avants et al. 2004) which will be briefly described here. It was ascertained during the initial experimentation and prototyping that Lagrangian registration, where the angiography-selected cine frame at phase *φ*_A_ (moving image) is directly registered to the perfusion-selected cine frame at phase *φ*_A_ (fixed image) is capable of providing reasonable quality only when the number of phases *n* is small, typically *n* < 4. In the Eulerian registration framework, the composite phase-to-phase transform *T*_C_, spanning the perfusion and angiography-selected phases *φ*_P_ and *φ*_A_, would be defined as follows:

(3) $$T_{\text{C}}=T\left(\varphi_{\text{P}},\varphi_{\text{P}+1}\right)⊗\ldots \;⊗T_{i,j}⊗\ldots ⊗T\left(\varphi_{\text{A}},\varphi_{\text{A}-1}\right),$$

where the indices [P,...,*i*, *j*,...,A] correspond to the cardiac frames from *φ*_P_ to *φ*_A_ in the cine series. The Eulerian approach to phase-to-phase cine registration proved to be an improvement over the Lagrangian approach during the initial prototyping and experimentation. However, the accuracy of registration was adversely affected by the accumulated registration error with the increasing number of phases, which in some cases spanned up to 80% of the total number of frames in the cine series.

In the work presented in this article the non-rigid 3D transform *T*_C_ between the *φ*_P_ and *φ*_A_ frames of the cine series is obtained through consecutive frame-to-frame 3D deformable registration of all phases from *φ*_A_ to *φ*_P_ as follows:

• The registration between the cine frames at phases *φ*_A_ (moving image) and *φ*_A−1_ (fixed image) is performed as a standard case of 3D deformable registration with a B-Spline transform.
• For all subsequent registration steps, the number of which is determined by the total number of frames from perfusion-selected frame *φ*_P_ to the angiography-selected frame *φ*_A_, the transform is initialised by the transform parameters from the preceding step.

The final phase-to-phase transform *T*_C_ obtained through consecutive registration of cine phases from *φ*_P_ to *φ*_A_ is defined as follows:

(4) $$T_{\text{C}}\;=\;R\left(\varphi_{\text{P}},\varphi_{\text{A}},T_{\text{R}}\left(\varphi_{\text{P}\;-1},\varphi_{\text{A}}\right)\right),$$

Where *R* is a generalised registration function of three inputs: fixed image, moving image and initial transform. In this case the term *T*_R_ (*φ*_P−1_, *φ*_A_) is the initialisation transform, defined recursively as follows:

(5) $$T_{\text{R}}=R_{n}\left(\varphi_{\text{P}-n},\varphi_{\text{A}},R_{n-1}\left(\varphi_{\text{P}-n-1},\varphi_{\text{A}},R_{n-2}\left(\ldots \right)\right)\right).$$

The phase-to-phase transforms were obtained with multi-resolution registration (two levels) with Mattes’ implementation of the MI image similarity metric, B-Spline transforms and a modified version of limited-memory Broyden, Fletcher, Goldfarb and Shannon optimizer (Byrd et al. 1995; Zhu et al. 1997). The phase-to-phase transforms were computed from the angiography-selected cardiac phase *φ*_A_ to the three distinct perfusion-selected phases, one for each perfusion slice location. The registration was performed for the longest required run of cine frames only once in sequential order, while the transforms for the specific perfusion-selected phases were recorded at the appropriate stages of the process. Both direct and inverse transforms were computed for each perfusion-selected phase because direct transforms are required for resampling images, while inverse transforms were required for transforming point sets.

### 4.5. The composite spatiotemporal transform

The composite transform *T*_M_ defined in Equation (1) includes three transform components described earlier: a rigid perfusion-to-cine transform *T*_P_, a non-rigid cine phase-to-phase transform *T*_C_ and a translation angiography-to-cine transform *T*_A_. The application of transform *T*_M_ to the angiography volume *I*_A_ results in its non-rigid warping into the cardiac phase of the corresponding perfusion frame: *I*_P _≅ *T*_M_.(*I*_A_). Thus, the coronary arteries captured in the angiography volume will be transformed along with all other features of the volume. However, if the segmentation of coronary arteries in the angiography volume is represented as a point set, then an inverse transform *T*_M_^−1^ is required in order to place the arteries into the perfusion coordinate space. The inverse transform $T_{M}^{-1}$ for n cine frames is defined as follows:

(6) $$T_{\text{M}}^{-1}=\;T_{\text{P}}^{-1}⊗T_{\text{C}}^{-1}⊗T_{\text{A}}^{-1},$$

where transforms *T*_P_^−1^, *T*_C_^−1^ and *T*_A_^−1^ represent the inverses of the rigid perfusion-to-cine, non-rigid cine phase-to-phase and translation angiography-to-cine transforms accordingly. The inversion of the rigid and translation transforms was obtained by the trivial inversion of the transform parameters. The inverses of all *T*_C_ transforms were computed at the same time with the direct transforms by swapping the fixed and moving images.

The transforms *T*_M_ and *T*_M_^−1^ provide spatial correspondence between the maximal contrast perfusion frames and angiography images which were acquired at distinct cardiac phases. Since the structure of the coronary tree is implicitly contained in the angiography images, the spatial transform (which maps angiography to perfusion images) is also applicable to any features which can be observed in the angiography volumes.

## 5. Motion correction in myocardial perfusion series

Myocardial perfusion analysis relies on the enhancement of signal intensity of the blood passing through myocardium, while the regions with diminished blood supply are observed as areas of lower signal intensity relative to the healthy parts of myocardium. Currently, the common method of myocardial perfusion assessment involves qualitative visual analysis of the series played as a movie loop. The regions of relatively reduced signal intensity are identified as perfusion defects caused by the obstructed blood flow.

In recent years, methods for quantitative and semi-quantitative measurement of myocardial blood flow (MBF) and myocardial perfusion reserve (MPR) have been developed in order to improve the diagnostic accuracy of myocardial perfusion imaging (Jerosch-Herold et al. 2004). These methods are based on the analysis of the signal-intensity curves which are also known as the dynamic contrast uptake curves. The signal intensity for each point in the curve is calculated from the regions of interest in the myocardium and the left ventricular blood pool; typically these regions are defined manually within each frame of the whole series.

Correction for motion artefacts during perfusion series acquisition is of critical importance because the intensity values must be derived from the spatially consistent myocardial regions to produce accurate results. Typically motion correction in perfusion series is carried out manually. Automated methods for motion correction in perfusion images have the potential to offer significant benefits by increasing the throughput and accuracy of perfusion analysis. However, the dynamic nature of cardiac MR perfusion series imposes strict time constraints on the acquisition protocols which lead to overall poor image quality and multiple problems with automated motion correction.

All three slices in a given frame of the dynamic perfusion series (basal, medial and apical) are acquired with a single heartbeat. The acquisition of the complete series during the first pass of the contrast agent through the heart usually takes between 30 and 60 heartbeats for rest series, and 40–80 heartbeats for stress series. The total acquisition time for either rest or stress series ranges from 45 to 60 s, which is too long to be performed in one breath-hold. As a result, the acquisition was done with one shorter breath-hold of 20–30 s preceded and followed by a series of shallow breaths. The timing of the breath-hold is intended to coincide with arrival of the contrast agent bolus in the left ventricle. In practice, however, patients are often unable to follow the instructions of the radiographer exactly; it is common to have perfusion series with very short breath-holds or deep breaths following the breath-holds. As a result, the images are often acquired with a wide variation of the diaphragm and heart positions. This problem is known as through-plane motion and its implications are examined in the next section.

The work of McLeish et al. (2002) reports that the magnitude of the respiration-induced motion of the heart is the largest in the trans-axial direction with smaller displacements in the left–right and anterior–posterior directions. The authors report respiration-induced translation of up to 23 mm combined with what appears to be rotations and non-rigid deformations. In the work of Milles et al. (2008), it is pointed out that through-plane motion violates the underlying principle of perfusion imaging which relies on the idea of tracking the concentration of the contrast agent for the same location over time.

In the literature on perfusion series registration, the respiration motion is often viewed as 2D motion. Small magnitudes of respiration motion are often treated differently from severe cases of through-plane motion, when in fact, patients’ respiration always causes some through-plane motion. In practice, the shape of the myocardium does not vary significantly around the medial slice. This may create an impression of the respiration motion occurring only in 2D without through-plane motion. The problem of respiration and through-plane motion cannot be solved perfectly with post-acquisition methods only. In light of the motion patterns associated with the current perfusion MRI protocols, all post-acquisition solutions for automated motion correction in perfusion series aim to offer a sensible compromise in order to achieve the best estimate for the perfusion-related parameters.

**Figure 5.**
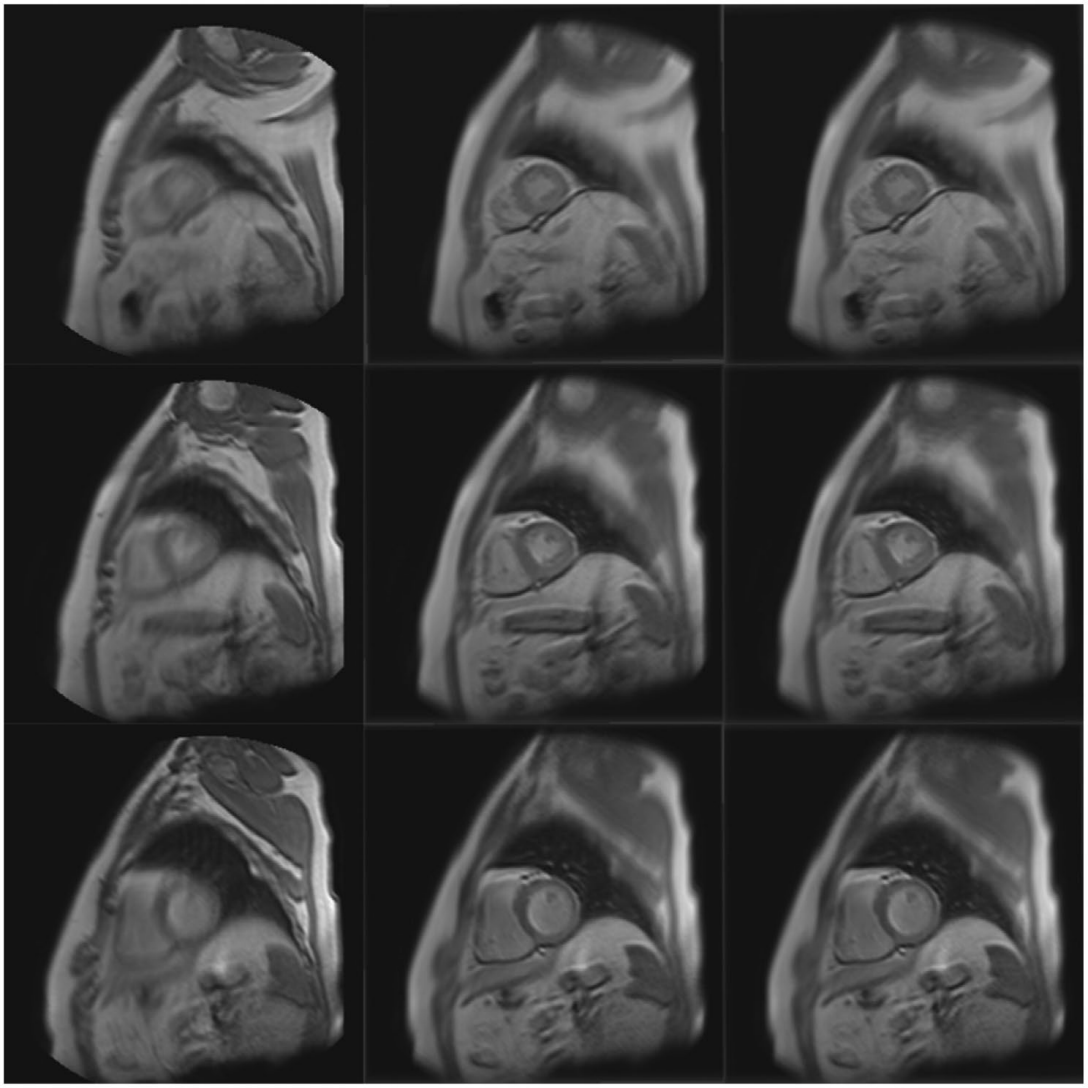
Evolution of average images for a stress perfusion series; rows: apical, medial and basal locations; columns: prior to correction, after translation and after rigid correction; the improvement of the clarity of the cardiac features from non-corrected average images to translation-corrected images supports the general observation that the bulk of the motion is compensated by translation correction.

The registration solution for motion correction in perfusion series presented here draws on the basic frame-by-frame registration approach reported by Li and Sun (2009). The key point of difference in the solution presented here from the basic frame-by-frame registration solutions is in the use of average frames as fixed images during registration; the use of average images draws on the ideas of group-wise registration published by Cootes et al. (2010). The rationale behind the use of average images is that they contain all features from the series. This makes them suitable to be used as the fixed images for the correction of slices from pre-contrast, right ventricular contrast, left ventricular contrast and contrast wash-out phases of the series as shown in Figure 5.

Another difference of the proposed solution lies in the physiologically informed approach to transform initialisation and transform parameter propagation to the below-basal locations. It was hypothesised that it might be sufficient to correct the motion only for the basal slice, and propagate the transforms to the medial and apical slices. This hypothesis is based on the following observations:

• Basal slices contain the highest information density within perfusion series; thus it is likely that motion correction parameters will be computed most accurately for basal slices within each series.
• The perfusion imaging protocol ensures that all three slices in each dynamic frame (basal, medial and apical) are acquired within 150 ms of each other in the same cardiac cycle. Since respiratory motion is relatively slow, the relative difference between the three slices in each frame can be considered negligible.

Motion correction was performed starting with translation-only registration, followed by 2D rigid correction. Apart from transform type, the rest of the registration assembly components were similar for translation and rigid stages. Here motion correction was achieved through multi-resolution registration (two levels) with a gradient descent optimizer and Mattes’ implementation of the MI image similarity metric.

## 6. Patient-specific coronary supply territories

Patient-specific assignment of coronary arteries to the corresponding blood supply territories in perfusion images is motivated by the need to determine the arteries responsible for perfusion defects. The method for patient-specific territories assignment introduced in this section fulfils this need. In addition, the coronary supply territories obtained with the method described here can be used directly in quantitative perfusion analysis. This method ensures that contrast uptake curves and perfusion-specific parameters such as MBF and MPR scores are computed for the physiologically coherent segments of the myocardium instead of the population-based AHA model of coronary blood supply.

The patient-specific territories were calculated for the maximal contrast frame in perfusion series on the basis of proximity of the coronary vessels warped into the spatiotemporal coordinate space of the corresponding perfusion frame/phase to the myocardium as it is observed in the given series.

In the context of this project, manual tracking of coronary arteries by cardiology experts was chosen for pragmatic reasons. The quality of the angiography data-sets acquired within the CE-MARC study was unsuitable for reliable automatic coronary artery segmentation/tracking. The angiography data-sets were acquired during free-breathing with a respiratory navigator. The respiratory navigator selects a subset of slices falling within a pre-specified range of a respiratory cycle; thus the images in a single temporal series belong to a range of closely clustered, but not identical, phases within the respiratory cycle. The respiratory navigator window in the CE-MARC angiography acquisition protocol was set to 5 mm, while coronary arteries range 2–4 mm in diameter. Under such circumstances gaps and discontinuities in the path of coronary arteries were frequently observed in the MRA data-sets from the CE-MARC study. The coronary arteries were annotated with an in-house tool developed for tracing centrelines of the coronary lumen in MR angiography data-sets; the annotations were examined by an experienced cardiologist.

**Figure 6.**
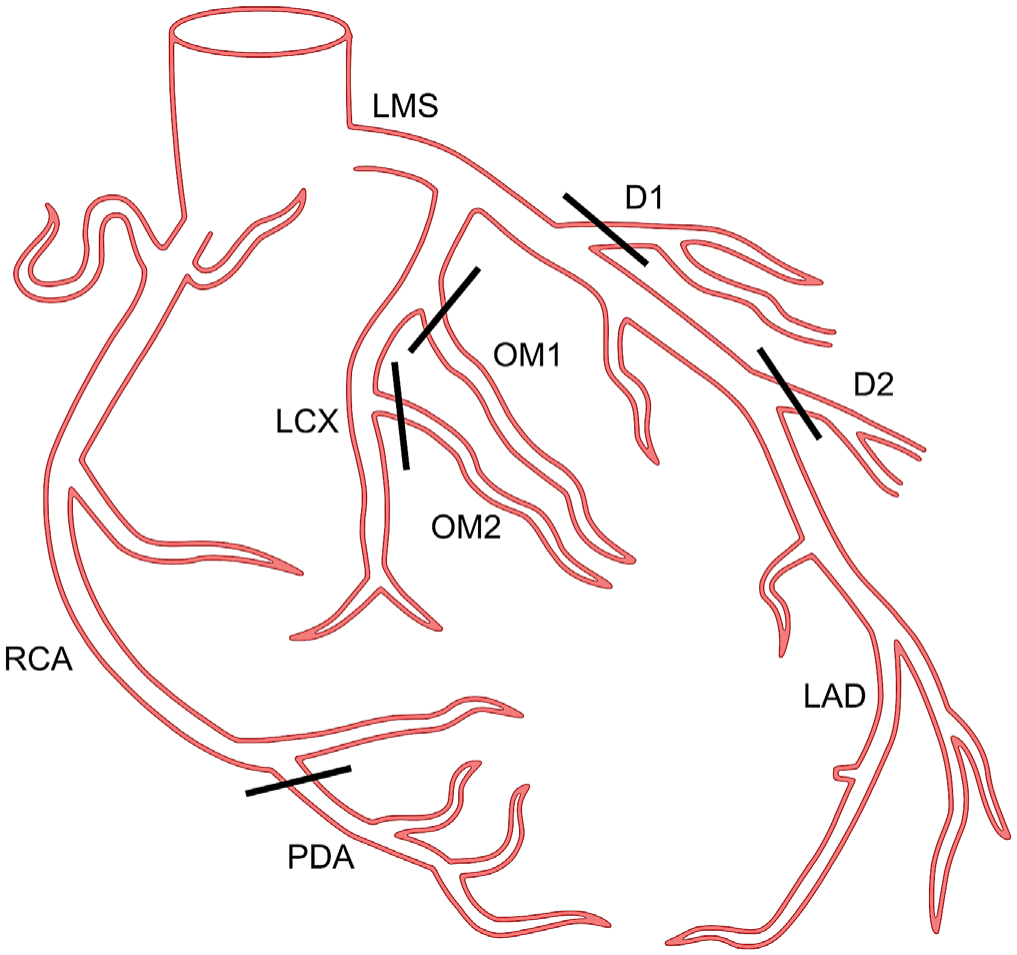
Coronary tree segments and their grouping during annotation: RCA group: right coronary artery (RCA), posterior descending artery (PDA); LAD group: left main stem artery (LMS), left anterior descending artery (LAD), first diagonal branch (D1), second diagonal branch (D2); LCX group: left circumflex artery (LCX), first obtuse marginal branch (OM1), second obtuse marginal branch (OM2).

A schematic representation of a coronary tree and its segments is shown in Figure 6. The segments of the coronary tree were allocated to the three coronary supply territories based on the major coronary tree branches: RCA, LAD and LCX. The centrelines of the visible branches were traced by an expert and examined by a cardiologist. In addition to the general low quality of the MRA data-sets, two types of specific problem were frequently encountered during annotation. First, in many cases the arteries could not be traced past the basal level of myocardium. Second, in some cases clearly visible vessels running along the expected paths for coronary arteries could not be classed as either arteries or veins. This was because of the abundance of vessel-like structures over the top of the LV where the branches must connect to the major LMS, LAD or LCX arteries.

**Figure 7.**
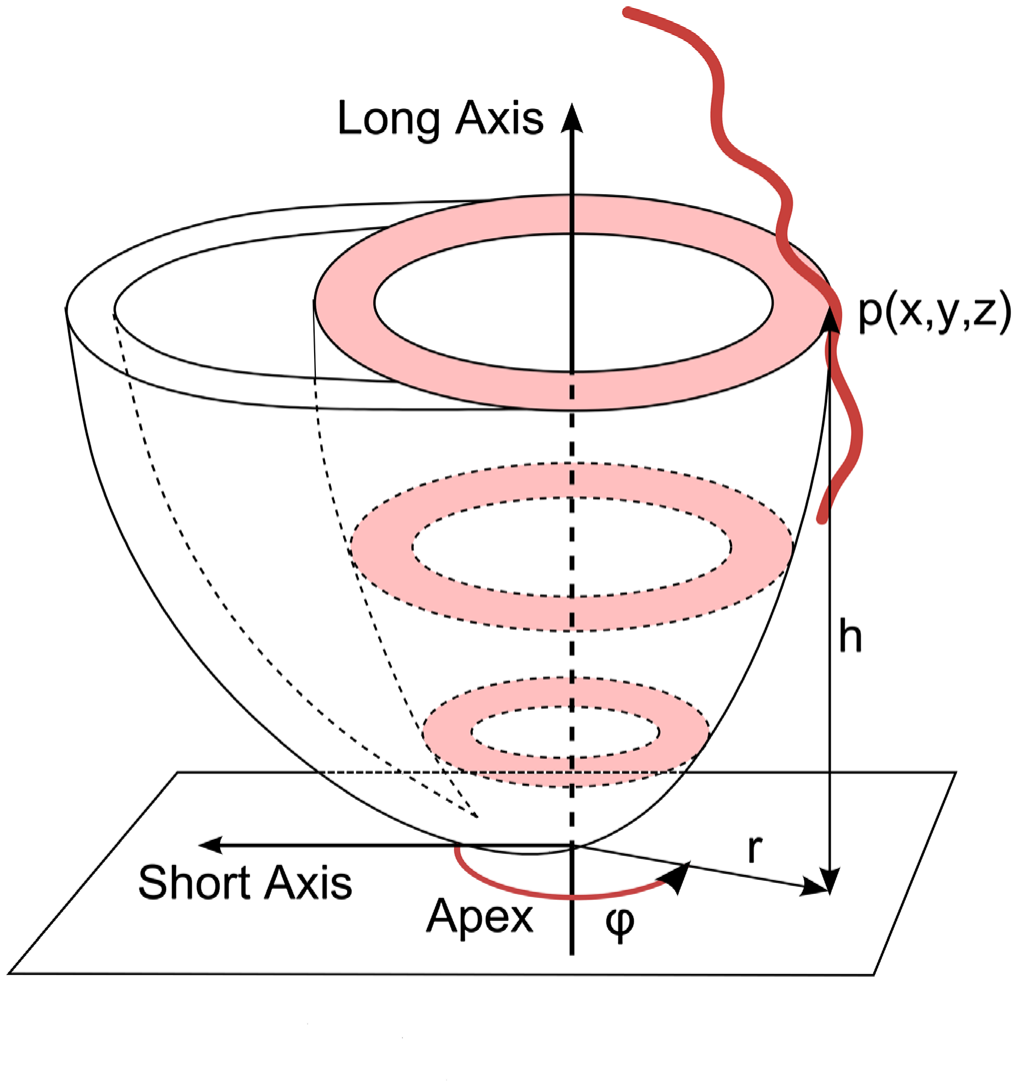
Parameterisation of points along segmented coronary path in polar coordinates for BEP generation; f is the angle *φ* between the SA vector and shortest distance vector r from point *p*(*x*, *y*, *z*) to the long axis passing through the apex; *h* is the distance from the point to the SA plane passing through apex.

A simplified method for conversion of 3D coronary trees to 2D representation was used in order to assess the quality of coronary tree annotations. The method for converting 3D coronary trees to 2D representation described here does not require the coronary tree to be projected on the segmented surface of the LV. As in the parameterisation of the myocardium in polar coordinates as presented by Termeer et al. (2010), any given point *p*(*x*, *y*, *z*) can be parameterised in polar coordinates as *p*(*φ*, *h*) as shown in Figure 7, where *φ* is the angle formed by the shortest distance vector *r* from the point to the left ventricular long axis passing through the apex and the SA vector; *h* is the distance from the point to the SA plane passing through the apex. Instead of projecting the coronary tree onto the segmented surface of the LV followed by the unwrapping of the 3D projection on the BEP, the plots were produced by interpreting the distance *h* and angle *φ* as 2D polar coordinates.

**Figure 8.**
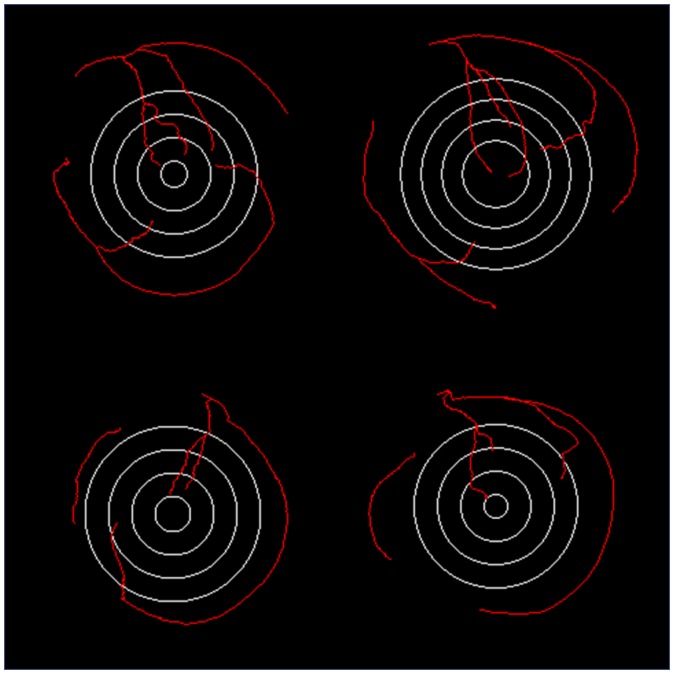
Examples of BEP-like visualisations of manually annotated 3D coronary trees plotted in 2D polar coordinates; the examples in (a) and (b) show the arterial trees from MRAs of good quality with common coronary anatomy (right-dominant); the plots in (c) and (d) are examples of less common coronary anatomy (left-dominant).

Figure 8 shows four examples of manually annotated coronary trees projected onto the BEPs. The basal, medial and apical bands along with the apex indicate the relative location of the basal, medial and apical slices from the corresponding perfusion series. For each plot, the basal slice from the key frame in perfusion series was registered to the angiography volume as described earlier. After registration, the distance *h* from the apex to the plane of the basal perfusion slice was calculated; this distance was used as the radius of the basal band. The outer and inner radii of the basal slice shown in the BEPs were calculated on the basis of the slice thickness in perfusion series. Similarly, the inner medial and apical bands were computed on the basis of the inter-slice offset. In perfusion series the variation of the size of the apical region in the plots is determined by the offset *h* along the long axis and the thickness of perfusion slices. For the cases where the perfusion slices were acquired further away from the apex, the apical region appears larger and *vice versa*.

BEPs provide a convenient visualisation of the coronary tree in 2D; this method of visualisation can be useful for comparative analysis of coronary anatomy. In the context of this work, the BEPs were used to select coronary tree annotations suitable for the computation of patient-specific coronary supply territories for the quantitative perfusion analysis. The criterion for selection was based on the presence of at least two major arteries at the medial level of the LV.

**Figure 9.**
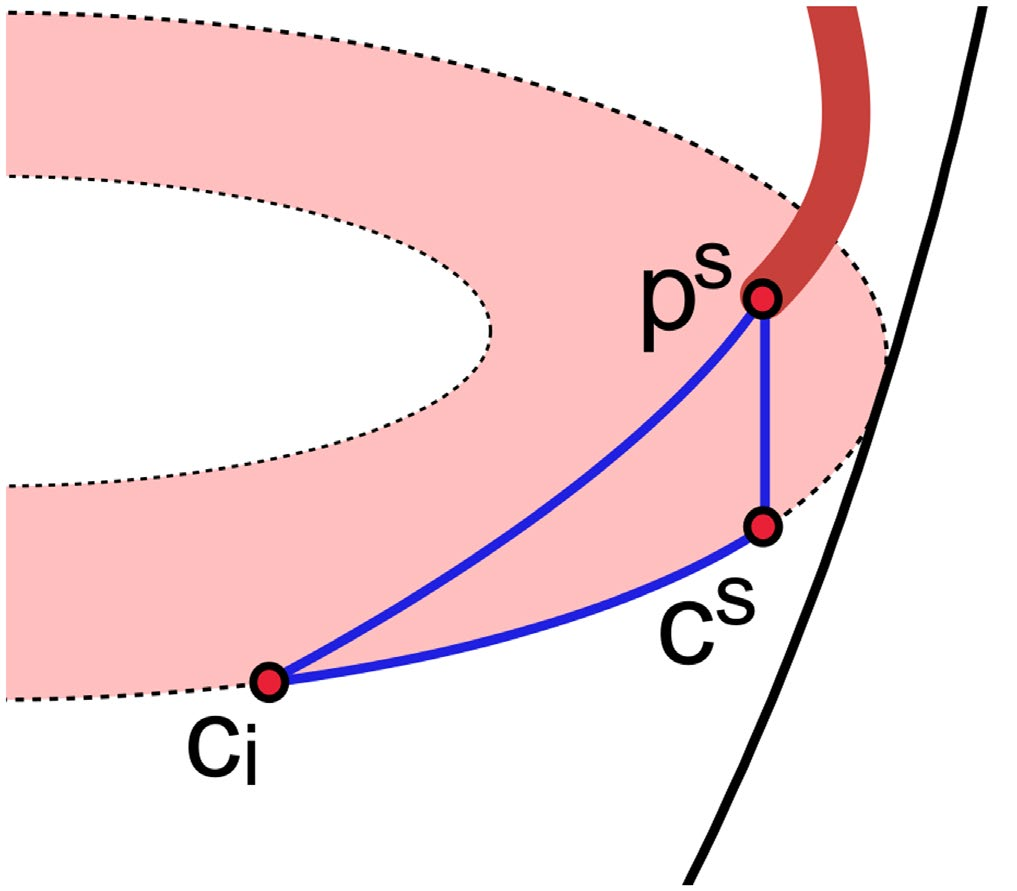
Calculation of a contour distance map for a single arterial segment: distance from *p*_*S*_ to *c*_*S*_ is shortest Euclidean distance from the given segment to the left ventricular endocardial contour; the length of the contour segment from *c*_S_ to *c*_*i*_ is the current running distance along the contour; the triangle *p*_*S*_, *c*_*S*_, *c*_*i*_ is wrapped around the surface of the LV approximated by an extruded cylinder-like shape defined by the left ventricular epicardial contour.

Along with the segmented coronary trees, the calculation of patient-specific territories also requires the contours following the left ventricular epicardial surface in the maximal contrast perfusion frame. Patient-specific territories for each of the basal, medial and apical locations were computed independently, because each perfusion slice location is associated with a different cardiac phase in the cine series. For a given slice location, the steps involved are as follows:

(1) Manually annotated points corresponding to a particular coronary tree were transformed using the corresponding inverse spatiotemporal transform spanning the angiography and perfusion phases of the cine series.
(2) Per-segment distance maps were calculated along the left ventricular epicardial contour for a given location for each of the arterial segments *s* ∈ {1..9}. The calculation of a segment-specific distance map for arterial segment starts with finding two closest points *p*_*S*_ and *c*_*S*_ from the set of segment points *P* *=* {*p*_1_ *… p*_*M*_} and the set of contour point *C* = {*c*_1_ *… c*_*N*_}, respectively, as shown in Figure 9. Distance along the contour for point *c*_*i*_ can be defined as follows:

(7) $$d^{S}\left(c_{i}\right)=\sqrt{\delta {\left(c_{i},c^{S}\right)}^{2}+{∥p^{S}-c^{S}∥}^{2}},$$

where *δ*(*c*_*i*_, *c* ^*S*^) is the accumulated distance between points *c*_*i*_ and *c* ^*S*^ along the contour and ||*p*_S_ −*c* ^S^|| is the shortest distance between the contour and closest point in the given arterial segment. This calculation is based on an assumption that near the plane of the contour, the surface of the ventricle can be approximated with a cylinder-like shape produced by the extrusion of the contour along the long axis of the LV. In this case, the right-angled triangle *c*_*i*_, *c* ^*S*^ and *p* ^*S*^ can be defined on the surface of the cylinder as shown in Figure 9. The segment-to-contour distance map is defined as follows:

(8) $$L^{S}={\left\{d^{S}\left(c_{i}\right)\right\}}_{i=1}^{N},$$

where $L_{i}^{S}$ refers to the point *ci* along the contour. A closed contour can be traced in both directions from point *c*_*S*_ while keeping track of the accumulated distances *δ*(*c*_*i*_,*c*_*S*_) for each of the two advancing points.

(3) For the computation of the RCA, LAD and LCX territories the segments *s* = {1…9} are grouped into *T* ^RCA^ {1, 2}, *T* ^LAD^ {3…6} and *T* ^LCX^ {7…9}. The territory-specific contour distance maps were calculated by combining the relevant segment-specific contour distance maps as follows:

(9) $$L^{T}=\left\{\text{min}{\left\{L_{i}^{S}\right\}}_{s\in T}\right\},$$

where *T* is either of *T* ^RCA^, *T* ^LAD^ or *T* ^LCX^ and $L_{i}^{T}$ is the distance value at point *c*_*i*_ along the contour.

(4) The territory-specific distance maps are combined into one contour label map by choosing the label for each point along the contour according to the minimum distance among all territory-specific distance maps:

(10) $$M=\{\underset{T}{\text{arg min}}\left\{L_{i}^{T}\right\}\}$$

(5) Next, Voronoi partitioning (Aurenhammer 1991) is obtained for the whole slice, where the contour label map *M* is used as input. The output of Voronoi partitioning has all points in the whole slice assigned to one of the three labels. The non-myocardium area of the slice is masked out by the left ventricular endocardial and epicardial contours which enclose the myocardium.
(6) Transforms obtained from respiratory motion correction in perfusion series were used to propagate the generated mask for the maximal contrast frame to the rest of the frames in the series. In this case, the inverse transforms were required in order to place the masks over the myocardium in the original perfusion images, instead of resampling perfusion images with the direct motion correction transforms.

**Figure 10.**
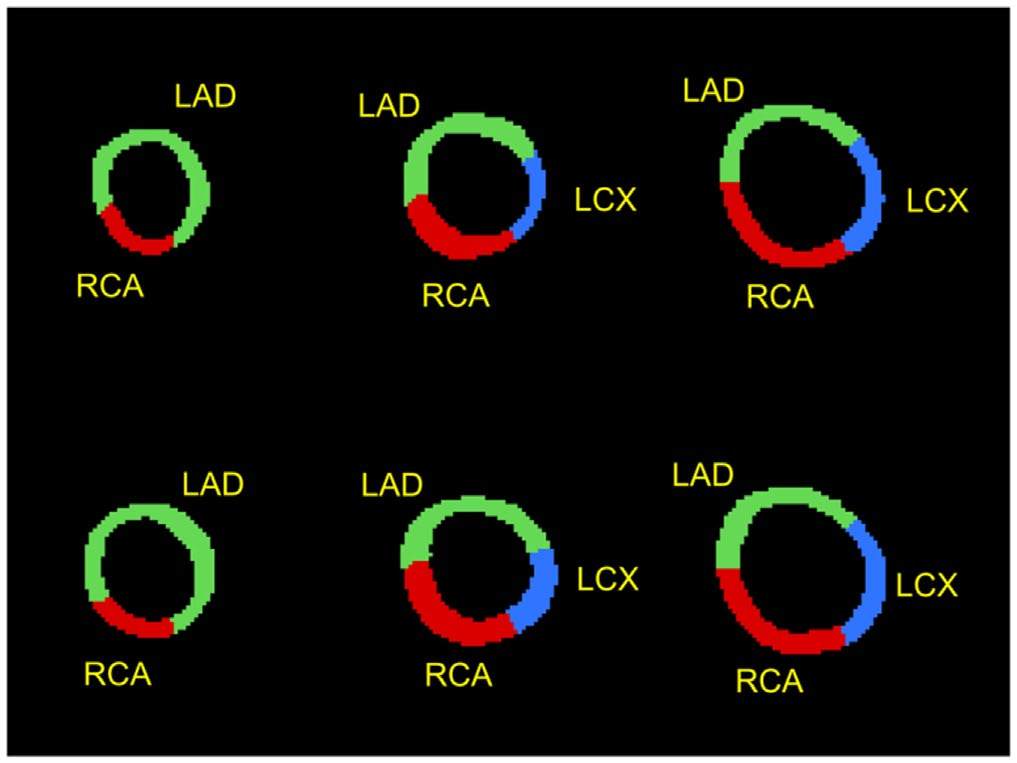
Examples of masks labelling patient-specific coronary supply territories; above: apical, medial and basal territories in rest series; below: the corresponding masks in stress series for the same patient; the variation of the thickness of the myocardium is due to the cardiac phase difference between corresponding slices.

Figure 10 shows an example of the masks defining patient-specific coronary supply territories for key frames in rest and stress series. With such masks, quantitative perfusion analysis can be carried out on patient-specific territories with the general perfusion quantification methods currently used with the AHA model of coronary blood supply. The masks for patient-specific coronary supply territories can be used in place of manually defined contours to identify the specific myocardial segments during perfusion quantification.

## 7. Evaluation methods

Evaluation was carried out in three separate steps, corresponding to the three main components of this work: mediated spatiotemporal registration, respiratory motion correction and the calculation of patient-specific territories. Although 50 clinical data-sets were available for evaluation, the evaluation steps which involved MRA whole-heart volumes were carried out in smaller numbers due to the low quality of some of the MRA data-sets which affected the reliability of manual segmentations. These limitations were applicable to the evaluation of the mediated spatiotemporal registration and the calculation of patient-specific territories. In the former case, the evaluation required manual segmentation of the left ventricular endocardial and epicardial surfaces, as well as the right ventricular endocardial surface. The blood-to-tissue interface can be segmented with the least ambiguity only in good quality MRA data-sets unaffected by blurring caused by patient motion. The difficulties associated with the manual coronary tree annotation for the calculation of the patient-specific territories imposed limitations on the number of data-sets suitable for the calculation of patient-specific territories.

### 7.1. Mediated spatiotemporal registration evaluation

The mediated spatiotemporal registration presented in this work was compared with a baseline method of perfusion-to-angiography registration reported by Hennemuth et al. (2008). The baseline perfusion-to-angiography registration method involved affine registration. For both methods, the accuracy of registration was evaluated on the basis of the Hausdorff distance metric (Huttenlocher et al. 1993) computed for manually defined contours in the perfusion series and the corresponding manual volumetric segmentations of the angiography volumes. For this evaluation, left ventricular endocardial and epicardial as well as right ventricular endocardial manual segmentations were carried out on 30 angiography 3D volumes (out of the 50 CE-MARC data-sets) where the clarity of the blood-to-tissue interface was suitable for manual segmentation. Similarly, left ventricular endocardial, left ventricular epicardial and right ventricular endocardial contours were delineated in the corresponding 30 rest and stress perfusion series.

The angiography-to-perfusion (rest and stress) transforms were computed with both methods; the resulting transforms were applied to the volumetric segmentations in order to transform them to the cardiac phase which corresponded to the appropriate maximal contrast perfusion frame and slice. The region of overlap of a perfusion slice with the corresponding transformed volumetric segmentation was used to define and extract a 2D slice containing the segmentation labels. Connected component analysis (Lehmann 2008) was used to extract the left ventricular endocardial, left ventricular epicardial and right ventricular endocardial contours from the corresponding 3D segmentation labels contained in the 2D slices. The Hausdorff distance values were calculated for each type of perfusion contours and the corresponding contours obtained from the volumetric segmentations.

### 7.2. Respiratory motion correction evaluation

The evaluation of respiratory motion correction in perfusion series was carried out on 50 perfusion data-sets from the CE-MARC study. The evaluation of the proposed respiratory motion correction was carried out in two sets of experiments.

The first set of experiments evaluated the accuracy of respiratory motion correction with a general method based on an object overlap metric. The experiments in this set involved only translation correction because of the translation-based manual motion correction applied to the reference contours. Translation motion correction was performed only for the basal slice and propagated to the medial and apical slices. After motion correction, the Dice coefficient values (Dice 1945) were calculated for left ventricular epicardial contours in all frames.

The second set of experiments compared the two variants for respiratory motion correction vs. manual motion correction in the context of quantitative perfusion analysis. In particular, the experiments were carried out with translation-based correction, rigid correction and deformable correction for all slices vs. the corresponding variants with basal-only correction with transform propagation. For each experiment, the set of motion correction transforms was recorded and converted to VDFs, with a resulting vector image for each slice and frame. It must be noted that the VDFs were obtained from the inverse transforms, because the deformation fields were used for transforming the key frame contours, rather than resampling the images. The derivation of inverse transforms for translation and rigid transforms was trivial; however, in the case of deformable motion correction the inverse transforms were obtained during registration, along with the calculation of direct transforms.

The VDFs were used to propagate the contours from the maximal contrast frame to the remaining times series. The resulting contours were used to generate signal intensity vs. time plot for the myocardium and LV blood pool. These curves were then converted to concentration values and analysed to generate MBF estimates using the methods described in the work of Biglands et al. (2011). The MPR scores were related to the coronary artery diagnosis derived from the reference standard, X-ray angiography. In order to evaluate the methods in terms of diagnostic accuracy, receiver operating characteristic (ROC) curves for manual and automatic respiratory motion correction in perfusion series were generated for each of the experiments and compared on the basis of the area under the curve (AUC) measure as per the method by DeLong et al. (1988).

### 7.3. Patient-specific territories evaluation

Out of the 50 data-sets from CE-MARC trial, only 18 coronary tree annotations were selected for this part of the evaluation. The selection criterion requiring at least two major coronary branches reaching the basal slice was set in order to obtain patient-specific coronary supply territories based on realistic instance of coronary anatomy.

The small size of the evaluation data-set – only seven diseased patients – does not allow for an extensive quantitative evaluation based on ROC curves analysis as was done for the evaluation of respiratory motion correction. The evaluation is based on the case-by-case diagnoses tables and qualitative comparison of the method for patient-specific coronary territories mapping to the AHA coronary blood supply model. In addition, visual examination of perfusion defects mapped to patient-specific territories was carried out to support the validity of the proposed approach.

## 8. Results

The evaluation results in this section are divided into three sections corresponding to the methods of evaluation described in the previous section.

### 8.1. Mediated spatiotemporal registration results

**Figure 11.**
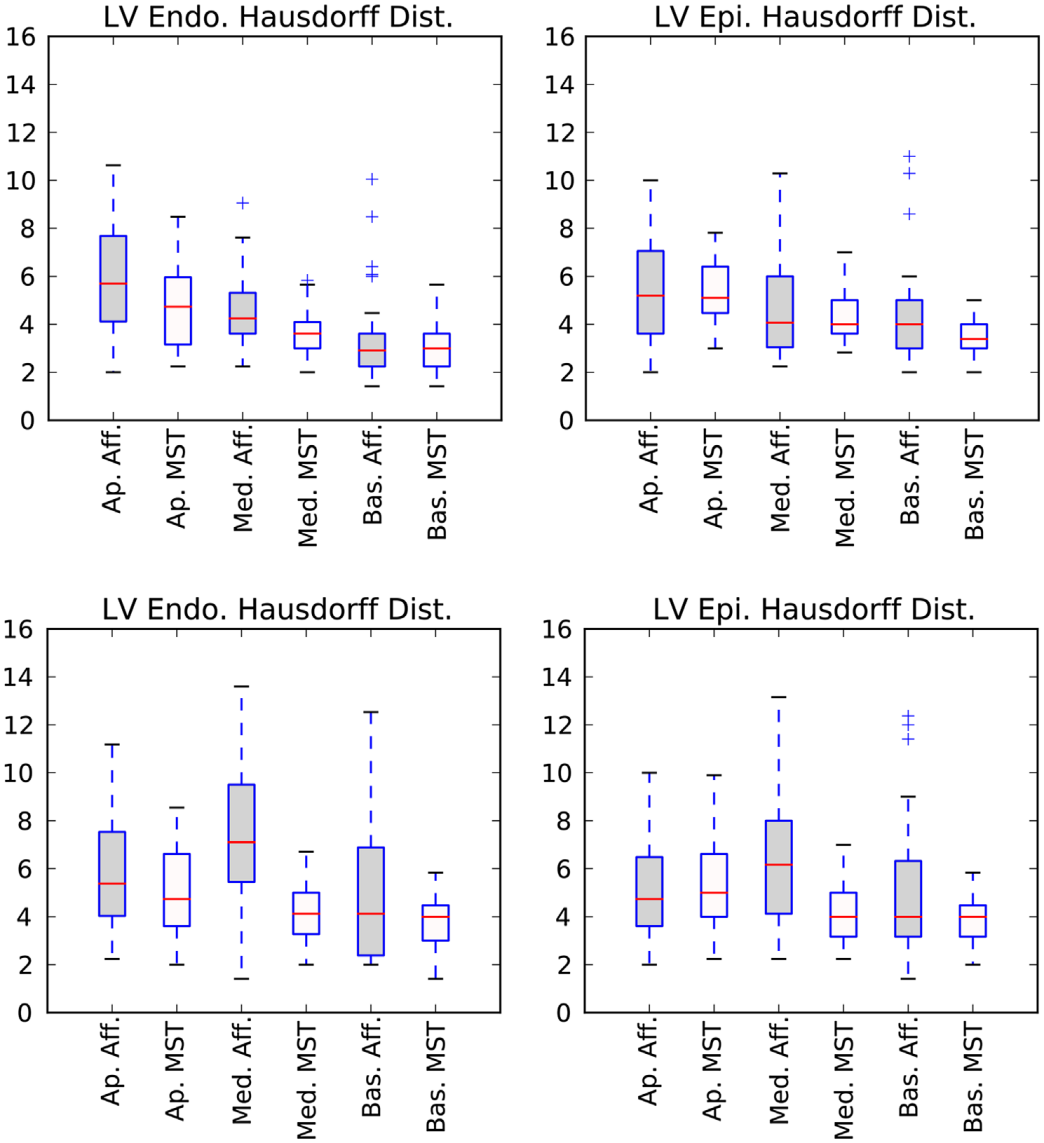
Comparison of the mediated spatiotemporal registration (MST) against the baseline approach on the basis of the Hausdorff distance metric for left ventricular endocardial and epicardial contours in rest (top) and stress (bottom) series for basal, medial and apical slices; Ap. – apical, Med. – medial, Bas. – basal, Aff. – affine registration, MST – mediated spatiotemporal registration.

The distributions of the Hausdorff distance values provided in Figure 11 display a strong trend favouring the accuracy of the mediated spatiotemporal registration. The most reliable results for both methods were achieved in the basal slices for the left ventricular endocardial contours, closely followed by the basal left ventricular epicardial, and then by the medial LV contours. Somewhat poorer performance was observed for the right ventricular endocardial contours, but this was not unexpected due to the difficulties associated with the right ventricle (RV) segmentation. The accuracy of the baseline method deteriorated for stress perfusion series to angiography. The most visible difference between the two methods is observed through the comparison of the Hausdorff distance values for left ventricular endocardial and epicardial contours for medial slices in stress perfusion series.

The summary of the Hausdorff distance metric values is provided in Table 1. The paired *t*-Test results indicate that a significant improvement in registration accuracy is achieved through the mediated spatiotemporal registration for medial left ventricular endocardial contours in rest perfusion series and medial left ventricular endocardial and epicardial contours in stress perfusion series.

**Table 1.** Results of paired-sample *t*-test which compared the Hausdorff distance metric for mediated spatiotemporal registration against the baseline registration method. A statistically significant improvement (*p* < *α*(0.05)) was observed for medial left ventricular endocardial contours in rest perfusion series and medial left ventricular endocardial and epicardial contours in stress perfusion series.

	LV endo.	LV epi.
Basal, Rest	No difference	No difference
Medial, Rest	*H*_d_: *t* = 3.386, *p* = 0.002	No difference
Apical, Rest	No difference	No difference
Basal, Stress	No difference	No difference
Medial, Stress	*H*_d_: *t* = 4.955, *p* = 2.9 × 10^−5^	*H*_d_: *t* = 3.507, *p* = 0.002
Apical, Stress	No difference	No difference

**Figure 12.**
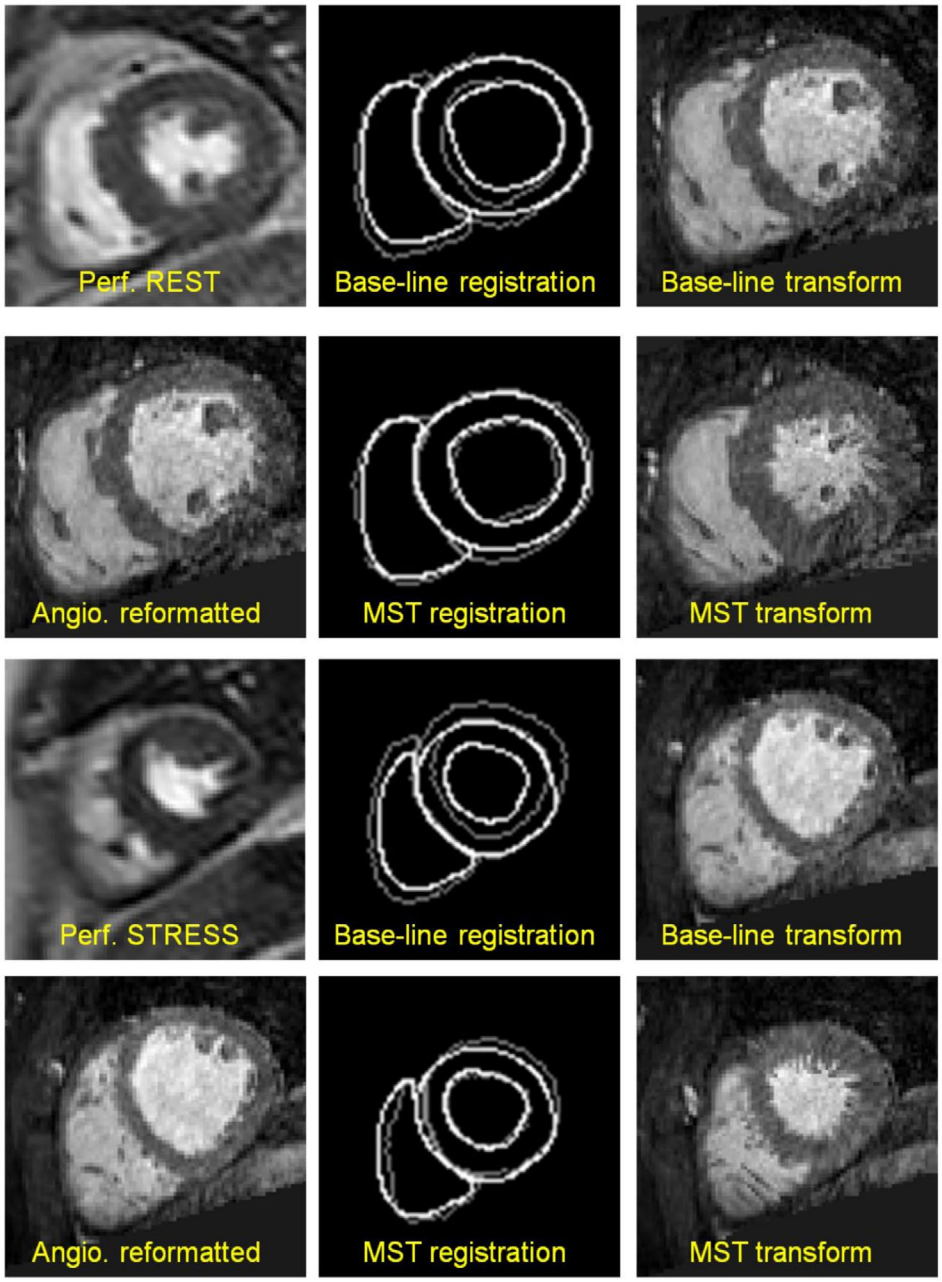
Examples of registration results for baseline correction and MST in medial perfusion slices: the first column in both figures contains key frames from perfusion series and the spatially corresponding reformatted slices from angiography volumes; the second column shows the contour overlap after baseline and the MST registration methods where perfusion contours are shown in white and the corresponding angiography contours are shown in grey; the third column shows the reformatted angiography images after the application of transforms obtained with the corresponding registration methods.

Examples of perfusion and angiography images with the corresponding contours shown in Figure 12 provide an illustrative comparison of the two registration methods. In these examples, the perfusion images show the myocardium in the systolic phase of the cardiac cycle. The rightmost column for both rest and stress examples shows the reformatted angiography image resampled after the baseline and the mediated spatiotemporal registration methods: the inferior performance of the baseline method is explained by an insufficient number of degrees of freedom in the affine transform used in the baseline registration method. On the other hand, the non-rigid phase-to-phase transform component *T*_*C*_ from the composite transform *T*_*M*_ derived through the mediated spatiotemporal registration method is a more appropriate geometric transform for solving the angiography-to-perfusion registration problem. In these examples, the mediated spatiotemporal registration outputs display a closer match between perfusion and angiography contours. Thickened myocardium in the transformed angiography images much closer resemble the original perfusion images.

### 8.2. Respiratory motion correction in perfusion series results

In each slice Dice coefficients were calculated for the manually defined left ventricular epicardial contour and the contour from the maximal contrast perfusion frame transformed with the inverse motion correction transform for this frame. A paired *t*-test between the Dice metric values summed for each slice suggests that the mean of the basal-only correction is lower than the all-slice correction variant both for rest (*μ*_1_ = 43.46, *μ*_2_ = 43.67, *t* = 3.72, *p* < *α*(0.05)) and stress (*μ*_1_ = 61.86, *μ*_2_ = 62.45, *t* = 3.47, *p* < *α*(0.05)) series. Although the mean error reduction does not offer much gain with the basal-only correction, the important finding here is that basal-only correction with transform propagation performs at least as well as the all-slice correction strategy.

**Figure 13.**
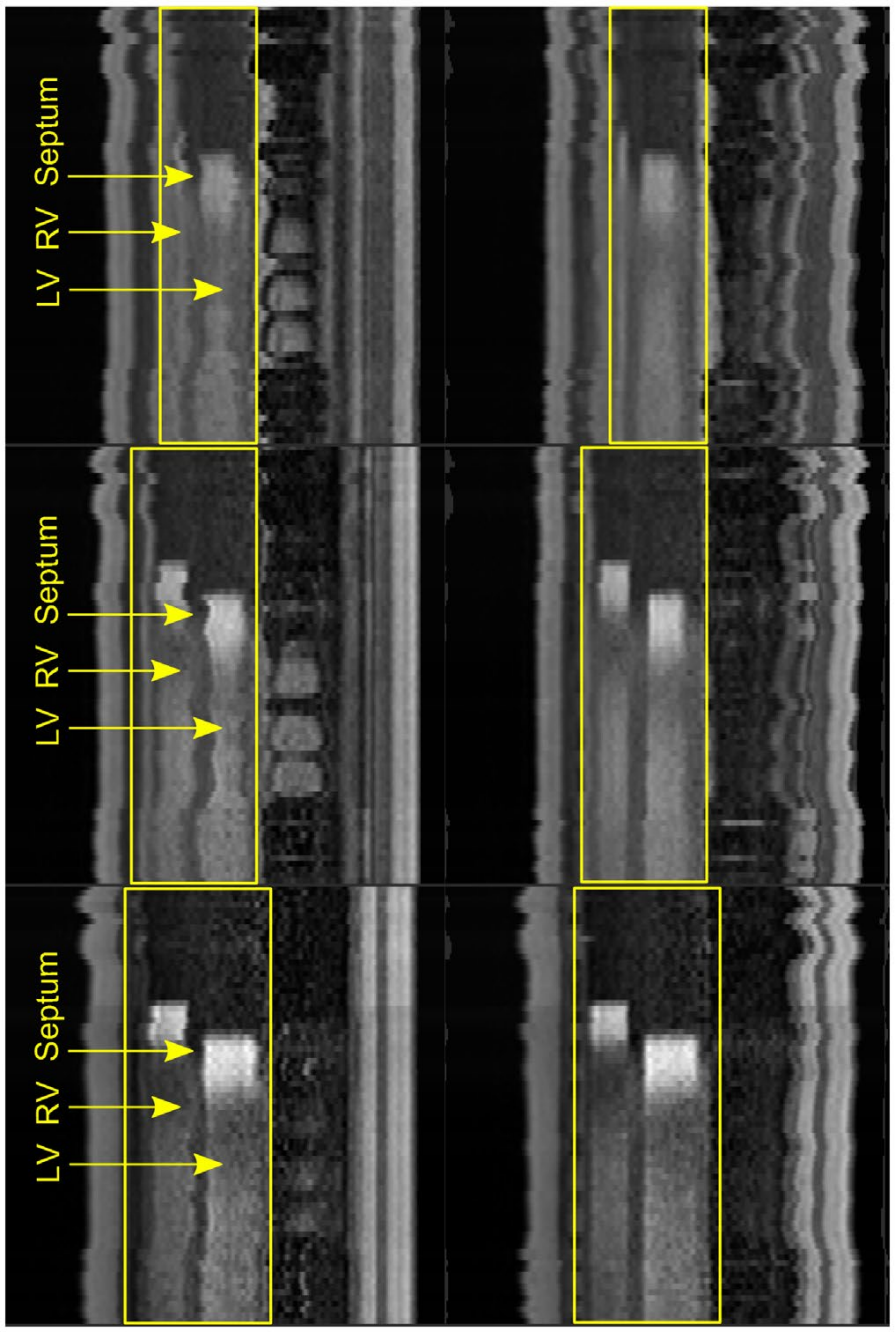
Stacked reformatted images for stress perfusion series; rows: apical, medial and basal locations; columns: before correction and after; the ROIs are shown with yellow boxes; within each ROI on the RV appears on the left, interventricular septum in the centre and the LV on the right; interventricular septum is the most obvious feature which can be used for visual validation of motion correction.

A visual example of respiratory motion correction is shown in Figure 13; the images were obtained by extracting reformatted slices along the XZ dimension from the 2D slices for a given spatial location stacked into 3D images. The extracted slices were defined to pass through the centre of manually defined ROIs to include the relevant cardiac features and contrast uptake events. The example shows the reformatted slices for the series before motion correction, after translation correction and after rigid correction. The main feature which can be used as a good indicator of motion observed in the original images and motion compensation in the corrected images is the interventricular septum. In addition, the images clearly show the pre-contrast, right ventricular contrast, left ventricular contrast and wash-out phases of the series. In the images prior to correction, the magnitude of respiratory motion can be observed from the changing position of the interventricular septum and ventricles, while the series after the first correction stage show that the bulk of motion has been recovered with translation transform. The series after the rigid correction stage in this example are very similar to the images after translation correction.

**Figure 14.**
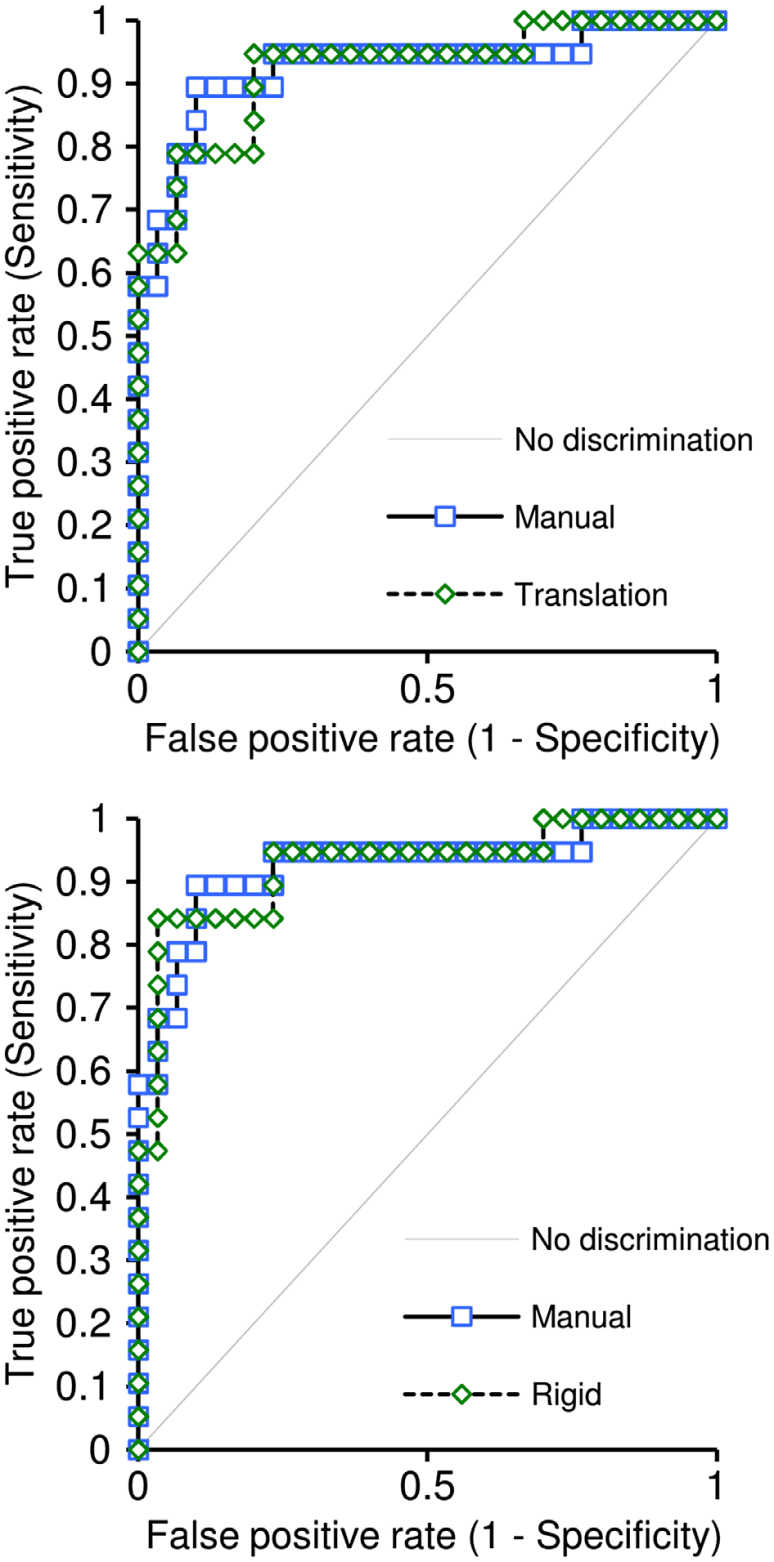
ROC curves for MPR scores generated for the two variants of respiratory motion correction in perfusion series: translation correction for basal slice with transform propagation and rigid correction for basal slice with transform propagation; the variants are compared to the ROC curve generated for the manual motion correction.

The comparison of the AUC values and confidence intervals (CIs) values for manual respiratory motion correction (0.93, [0.84, 1.00]) against the AUCs and CIs for both variants of the evaluated respiratory motion correction (translation: 0.92, [0.84, 1.00]; rigid: 0.93, [0.84, 1.00]) indicate that diagnostic accuracy of quantitative perfusion analysis carried out with perfusion series after automated motion correction is equivalent to the diagnostic accuracy of perfusion analysis with manual respiratory motion correction (Figure 14).

### 8.3. Patient-specific coronary supply territories results

For a total of 54 coronary arteries, eight cases (15%) were diagnosed incorrectly with the AHA model, while only five cases (9%) were diagnosed incorrectly with perfusion analysis based on patient-specific coronary supply territories as summarised in Table 2. The results of perfusion quantification with patient-specific coronary territories for the RCA were less reliable than for the LAD and LCX artery.

**Table 2.** Fraction of coronary arteries correctly diagnosed with ischaemia with the AHA model and patient-specific territories (PST).

RCA		LAD		LCX	
AHA	PST	AHA	PST	AHA	PST
16/18 (89%)	14/18 (78%)	14/18 (78%)	17/18 (94%)	16/18 (89%)	17/18 (94%)

**Figure 15.**
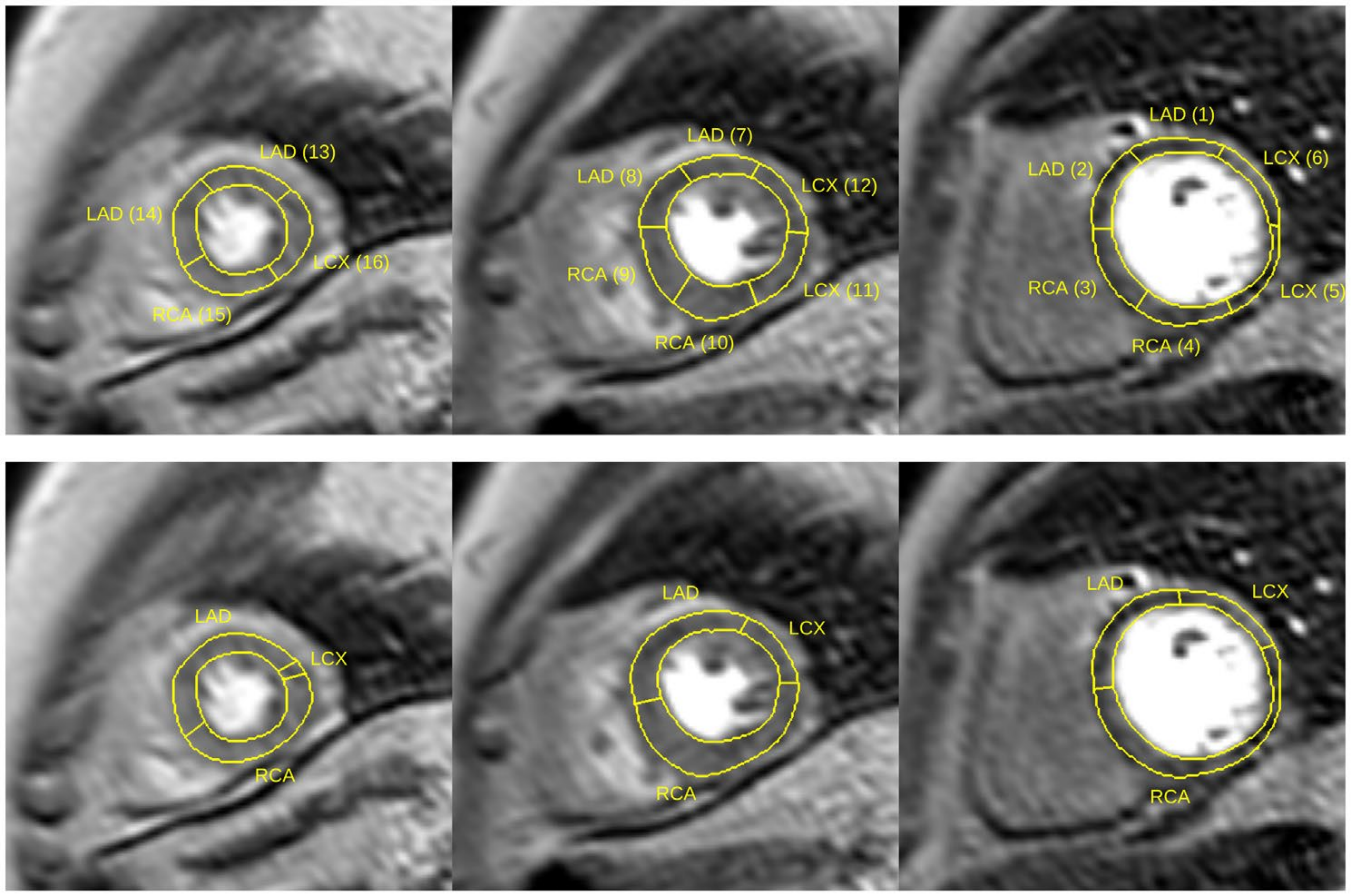
Comparisons between the AHA model (top) and patient-specific territories (bottom) for a patient with a right-dominant coronary supply system.

**Figure 16.**
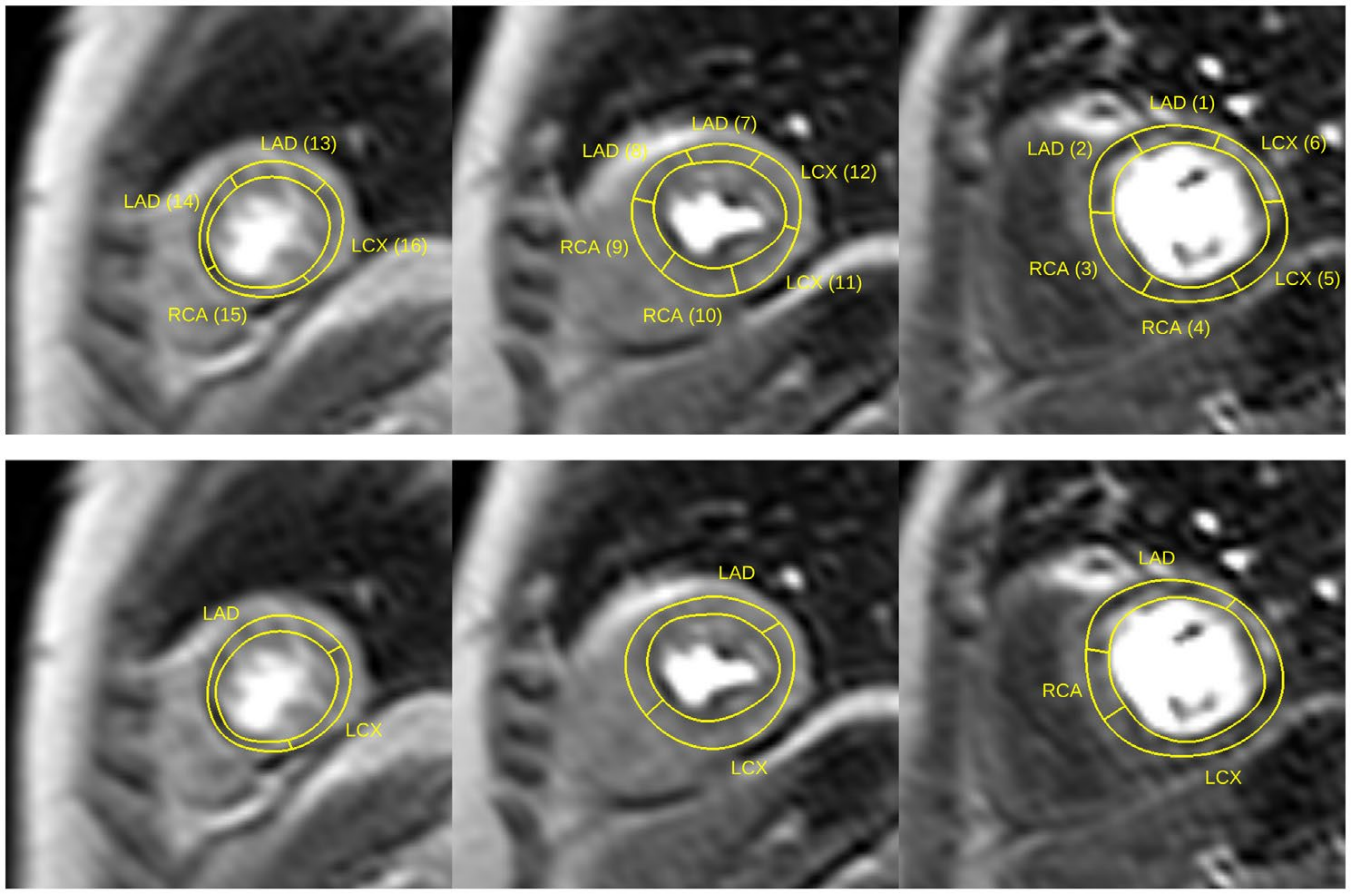
Comparisons between the AHA model (top) and patient-specific territories (bottom) for a patient with a left-dominant coronary supply system.

The patient-specific territories were markedly different from the AHA model as illustrated in Figures 15 and 16. In the case of a right-dominant system shown in Figure 15 (with the corresponding BEP shown top-left Figure 8) the RCA territory appears underestimated in all three slices according to the AHA model. The case presented in Figure 16 (with the corresponding BEP shown bottom-right Figure 8) provides another example of a left-dominant system where the LCX artery reaches to the inferior segments of the AHA model. As a result, the LCX territory appears grossly overestimated in the AHA model. The apical slices are shown in all examples for completeness, although the limitations of coronary tree annotation listed earlier make the mapping of coronary supply territories in the apical slices less reliable than in the medial and basal slices.

## 9. Conclusions and discussion

This article has presented a method for the registration of coronary arteries to perfusion series in combination with quantitative perfusion analysis. To our knowledge, this work presents for the first time a method of patient-specific perfusion analysis and evaluates perfusion registration in terms of its diagnostic accuracy. The experiments described in this work have shown that it is possible to derive patient-specific coronary supply territories from MRA and that these territories depart from the territories defined by the AHA model of coronary blood supply. The main contribution of this work is the proof of concept that patient-specific coronary anatomy in MRA can be used to provide a more accurate estimate of coronary supply territories in comparison to the coronary territories defined by the AHA model. In addition, this work shows that automated respiratory motion correction in perfusion series is capable of providing diagnostic accuracy comparable to manual motion correction.

The AHA model assumes proportionate contribution from all coronary arteries, which is the best assumption in the absence of other evidence. However, the described method for deriving patient-specific territories incorporates into the calculation the exact location of the coronary arteries and their proportionate contribution to the blood supply based on the proximity to myocardial tissue.

The AHA model relies on the definition of the RV insertion point which determines the position of the first segment and the relative location of the other segments. The AHA recommends that the RV insertion point is identified at the site of attachment of the right ventricular wall to the anterior part of the LV; this method of defining insertion points was used in this work. However, it is also common to place the insertion point at the attachment site of the right ventricular wall to the inferior part of the LV. This method of defining insertion points was used in this work. The site of attachment is often difficult to pinpoint and introduces variability in the positioning of the AHA model segments. Patient-specific territories circumvent this inconsistency by eliminating the need to define insertion points altogether.

DCE-MRI images are acquired at different phases of the cardiac cycle, while MRA is usually acquired at the end-diastolic part of the cycle. The deformable phase-to-phase transform component of the mediated spatiotemporal registration is required for recovering rotational motion between systolic and diastolic phases as well as phase-to-phase contractile motion. Both types of motion affect the position of coronary arteries. In the instances of the systolic-to-diastolic phase difference, when the myocardium is shown fully contracted in medial perfusion slices, the phase-to-phase deformable transformation component ensures that the coronary arteries remain close to the surface of the myocardium, which is important for the accurate calculation of patient-specific coronary supply territories.

It is generally acknowledged that the AHA model provides a suitable approximation for mapping the results of X-ray angiography onto myocardial anatomy in a consistent way in the absence of a more exact method. However, patient-specific models derived from MR or CT angiography have the potential to replace the AHA model with the mapping based on the actual coronary anatomy. The use of patient-specific coronary supply territories has the potential to enhance perfusion analysis by providing a mechanism for computing quantitative perfusion parameters for the artery-specific regions of the myocardium.

It is acknowledged here that the ‘true’ coronary supply territories cannot be obtained with the method described in this work because the microvasculature supplied by a given coronary artery might contribute to the territories of the adjacent artery. This can be observed in the ischaemic patients where adjacent arteries compensate for the lack of blood supply in the territories normally supplied by a stenosed artery via the formation of collateral vessels. Collateral arteries in the coronary arterial supply system compensate for hypoperfusion caused by the poor state of one or more of the main coronary arteries (Cicutti et al. 1994). Nonetheless, the method for obtaining patient-specific coronary supply territories described in this work offers an improvement over the AHA coronary supply model.

A further improvement of this work could be achieved by breaking up the grouping of the smaller arteries into the sub-territories of the RCA, LAD and LCX artery. In this case, the coronary supply territories could be calculated on a per-branch basis to provide a finer picture of the coronary blood supply. A significant stenosis of any of the branches of the coronary tree can lead to ischaemic heart disease. Thus, in theory, it could be beneficial to produce per-branch patient-specific coronary supply territories. In the case of the research reported in this work, the limited quality of the angiography data from the CE-MARC trial, and that from MR angiography in general, could support only the coarsest level corresponding to the RCA, LAD and LCX blood territories. The smaller branches of the coronary tree remained undetected in many of the MRA data-sets even though they were clearly visible in X-ray angiography.

The limited quality of MRA data-sets used in this study poses a limitation on the method for calculating the patient-specific coronary supply territories. X-ray is considered the gold-standard for the imaging of coronary arteries and detection of stenosis. This work can be extended with one of the methods for reconstructing 3D arterial trees from multi-plane X-ray angiography as previously reported by Zifan et al. (2008) and Cardenes et al. (2012). However, the invasive nature of X-ray angiography procedures suggests a preference for non-invasive sources of angiography images. The continuing improvement in the quality of MRA has the potential to improve the reliability of the proposed method for the computation of patient-specific territories. Currently the quality of CTA is superior to MRA; the method for computing patient-specific coronary supply territories described in this work could be used with CTA directly without modifications. With improved MRA, an automated method for vessel tracing and segmentation could be used to replace the time-consuming manual annotation of coronary arteries, which would further improve the presented work.

## Disclosure statement

No potential conflict of interest was reported by the authors.

## Funding

This work was supported in part by the Top Achiever Doctoral Scholarship awarded by the Tertiary Education Commission of New Zealand [grant number UOLX08001]; Clinical data were obtained within the CE-MARC study funded by the British Heart Foundation Grant [grant number RG/05/004].
